# From duplication to divergence: Single-cell insights into transcriptional and cis-regulatory landscapes in soybean

**DOI:** 10.1093/plcell/koaf279

**Published:** 2025-11-24

**Authors:** Xiang Li, Xuan Zhang, Robert J Schmitz

**Affiliations:** Department of Genetics, University of Georgia, Athens, GA 30602, USA; Department of Genetics, University of Georgia, Athens, GA 30602, USA; Department of Genetics, University of Georgia, Athens, GA 30602, USA

## Abstract

Gene duplication is a major source of evolutionary innovation, enabling the emergence of novel expression patterns and functions. Leveraging single-cell genomics, we investigated the transcriptional and cis-regulatory landscapes of duplicated genes in cultivated soybean (*Glycine max*), which has undergone 2 rounds of whole-genome duplication. Our analysis revealed extensive diversity of transcriptional profiles within and across tissues among duplicated gene pairs. Within-tissue divergence was largely attributable to genetic variation in their associated accessible chromatin regions (ACRs), where cis-regulatory elements reside, whereas cross-tissue divergence was more likely shaped by dynamics in ACR chromatin accessibility profiles across tissues. Distinct duplication mechanisms also likely give rise to different types of cis*-*regulatory variants, contributing variably to transcriptional divergence. By comparing ACRs associated with gene sets derived from 2 rounds of whole-genome duplication and sharing a common ancestral gene, we found that most ACRs retained one or multiple corresponding duplicated sequences in which mutations gradually accumulated over time, while a subset likely arose de novo. Finally, we traced the evolution of cell-type-specific expression and cell-type-specific ACRs within duplicated gene sets, illustrating a powerful framework for identifying candidate regulatory regions driving cell-type-specific expression. Collectively, our findings highlight the important role of cis*-*regulatory evolution in shaping transcriptional divergence in a spatiotemporal manner, uncovered with the resolution of single-cell genomics.

## Teaser

Differences in regulatory DNA shape gene expression diversity of duplicated genes in soybean, with patterns across cells within a tissue and across tissues, uncovered using single-cell genomics.

## Introduction

Gene duplication is a crucial driver of evolution, providing the raw materials for new gene functions and expression patterns (Lynch and Conery 2000; Birchler and Yang 2022). It arises through small-scale duplication, such as tandem or proximal duplication, or through whole-genome duplication that replicates entire genomic segments simultaneously (Zhang 2003; Kuzmin et al. 2022). These mechanisms generate duplicated genes with varying evolutionary rates, essentiality (e.g. whether a gene is required for survival or normal growth), and functions (Freeling 2009; Qiao et al. 2019; Shi et al. 2020; Cai and Des Marais 2024; Benoit et al. 2025). In plants, a large proportion of annotated genes have duplicates, mostly derived from whole-genome duplication, consistent with the prevalence of paleopolyploidy in land plants (Wendel 2000; Blanc and Wolfe 2004; Panchy et al. 2016; Tiley et al. 2016). The retention of these duplicates has been instrumental in shaping plant genomes and contributing to important agricultural traits (Panchy et al. 2016; Salman-Minkov et al. 2016; Benoit et al. 2025).

Retained duplicates may be subject to relaxed selective constraints, with one or both copies sometimes acquiring new functions (neofunctionalization) that contribute to adaptation or partitioning ancestral functions (subfunctionalization). However, functional divergence is not always necessary for their retention and typically unfolds over extended evolutionary timescales (Panchy et al. 2016; Birchler and Yang 2022). Multiple mechanisms have been proposed to account for the dispensability of retained duplicates (Innan and Kondrashov 2010; Panchy et al. 2016; Qiu et al. 2020). Moreover, factors such as selection and genetic drift can also influence gene retention (Panchy et al. 2016; Iohannes and Jackson 2023). On the other hand, this redundancy invariably complicates genotype–phenotype associations and limits the transferability of genetic findings across closely related species (Iohannes and Jackson 2023; Benoit et al. 2025). Despite their evolutionary and functional significance, the dynamics of gene duplication and diversification over short evolutionary timescales remain insufficiently explored (Benoit et al. 2025).

Divergent expression between duplicated genes is a widespread phenomenon and provides crucial insight into their evolution. In Arabidopsis, 70% of duplicated gene pairs exhibit significant transcriptional differences (Ganko et al. 2007). Similarly, approximately 50% of soybean duplicated genes, derived from whole-genome duplication, show transcriptional divergence (Roulin et al. 2013). This divergence is likely driven by genetic variants within regulatory regions (Wray et al. 2003; Tran et al. 2024), particularly cis-regulatory elements that control spatiotemporal expression patterns (Wittkopp and Kalay 2012; Schmitz et al. 2022). In cotton, divergence in cis-regulatory sequences between its diploid progenitors contributes to transcriptional divergence in 40% of the duplicated genes (Chaudhary et al. 2009). Similar patterns have been observed in Arabidopsis (Haberer et al. 2004), soybean (Fang et al. 2023), strawberry (Fang et al. 2024), and additional cotton studies (Han et al. 2022; Hu et al. 2024). Despite these valuable insights, an underexplored question remains: what forms of transcriptional divergence and which types of cis-regulatory variation are maintained in duplicated genes?

Soybean (*Glycine max*) having gone through 2 rounds of whole-genome duplication, approximately 59 and 13 million years ago (Schmutz et al. 2010; Koenen et al. 2021; Zhuang et al. 2022), makes it an excellent model for exploring this question. Gene pairs resulting from both whole-genome and small-scale duplication have been identified in soybean (Qiao et al. 2019). Previous studies have also explored the evolutionary fates of duplicated genes in soybean, highlighting divergence in gene expression and the role of cis-regulatory control (Du et al. 2012; Zhao et al. 2017; Fang et al. 2023). However, these studies primarily assessed transcriptional divergence at the tissue level, often relying on differential expression as the sole metric (Fang et al. 2023), which likely oversimplifies “divergence” and overlooks differences at the cellular level. For instance, even when 2 duplicated genes exhibit similar overall expression levels within a given tissue, they may display distinct expression patterns across different cell types within that tissue. This raises a critical question: do tissue-level and magnitude-based comparisons adequately capture transcriptional divergence, and to what extent does genetic variation in cis-regulatory regions drive such differences? Duplicated genes, which often retained copies of their associated regulatory regions, offer a powerful model for dissecting these regulatory relationships. Revealingly, variation in these regions could also result in cell-type-specific expression divergence, offering additional insight into the regulatory basis underlying cellular specificity and informing potential applications in synthetic biology. The recent availability of single-cell assay for transposase-accessible chromatin sequencing (scATAC-seq) and single-nucleus RNA sequencing (snRNA-seq) data of different soybean tissues (Zhang et al. 2025) enables the investigation of transcriptional and cis-regulatory divergence at single-cell resolution in a spatiotemporal context.

In this study, we integrated single-cell expression and chromatin accessibility profiles to examine transcriptional and cis-regulatory dynamics of duplicated genes within and across tissues. This approach provides a more nuanced understanding of transcriptional divergence and its correlation with cis-regulatory sequence variation, chromatin accessibility, and gene duplication mechanisms. Given that cis-regulatory elements are typically co-duplicated with genes during whole-genome duplication, we further examined their evolutionary trajectories and contributions to expression divergence in gene sets derived from 2 rounds of whole-genome duplication and sharing a common ancestral gene. Our analysis revealed that most cis-regulatory elements retained one or multiple corresponding sequences across the duplicated genes, whereas a subset likely evolved de novo. Finally, we explored the evolution of cell-type-specific expression within duplicated gene sets and the dynamics of their associated cis-regulatory regions, exemplifying how integrating cell-type specificity with regulatory sequence conservation to identify candidate regulatory regions driving cell-type-specific expression. Overall, our findings underscore the power of single-cell genomics in revealing the divergence of expression and chromatin accessibility in duplicated genes, offering key insights into the evolution of cis-regulatory regions and gene duplication.

## Results

### Distinct within-tissue expression patterns of duplicated gene pairs uncovered by single-cell genomics

**Figure 1.**
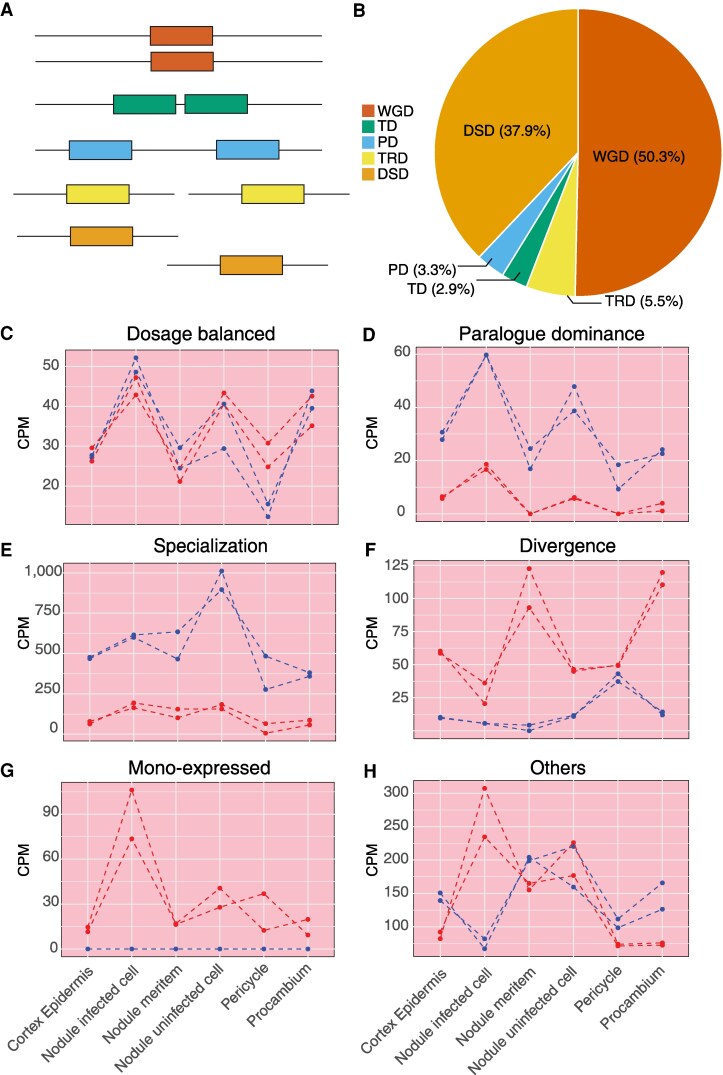
Different duplication mechanisms and distinct within-tissue expression patterns. **A)** Illustration of different gene duplication mechanisms, including whole-genome duplication (WGD), dispersed duplication (DSD), proximal duplication (PD), tandem duplication (TD), and transposed duplication (TRD). **B)** Percentages of duplicated gene pairs derived from different duplication mechanisms. **C** to **H)** Representative examples of duplicated gene pairs exhibiting distinct within-tissue expression patterns in nodule tissue: **C)** dosage balanced, **D)** paralogue dominance, **E)** specialization, **F)** divergence, **G)** mono-expressed, and **H)** others. In each panel, blue and red dots/lines represent the 2 genes within a duplicated pair, with 2 biological replicates included for each gene.

We identified 59,351 duplicated gene pairs in soybean using the DupGen_finder pipeline (https://github.com/qiao-xin/DupGen_finder), with common bean (*Phaseolus vulgaris*) as the outgroup. These pairs were classified into 5 categories: whole-genome duplication and 4 small-scale duplication, including tandem duplication (closely adjacent duplicated genes on the same chromosome), proximal duplication (duplicated genes located nearby on the same chromosome but separated by up to 10 other genes), transposed duplication (duplicated genes transposed to distant genomic positions, with the ancestral copy being either intra- or inter-species colinear), and dispersed duplication (duplicated genes scattered across the genome, neither neighboring nor colinear) (Fig. 1A and Supplementary Table S1). Importantly, because a single gene can experience multiple duplication events through distinct duplication mechanisms (e.g. a gene originating from whole-genome duplication may later be tandemly duplicated), we allowed redundancy in our classification, that is, the same gene may be represented in more than one duplication categories across different gene pairs. Among all identified gene pairs, whole-genome duplication derived gene pairs represented the largest proportion (50.3%), followed by dispersed duplication (37.9%) (Fig. 1B and Supplementary Table S2). The remaining 12% of duplicated gene pairs arose from tandem, proximal, and transposed duplication, with transposed duplication being the most prevalent among them (Fig. 1B and Supplementary Table S2).

**Figure 2.**
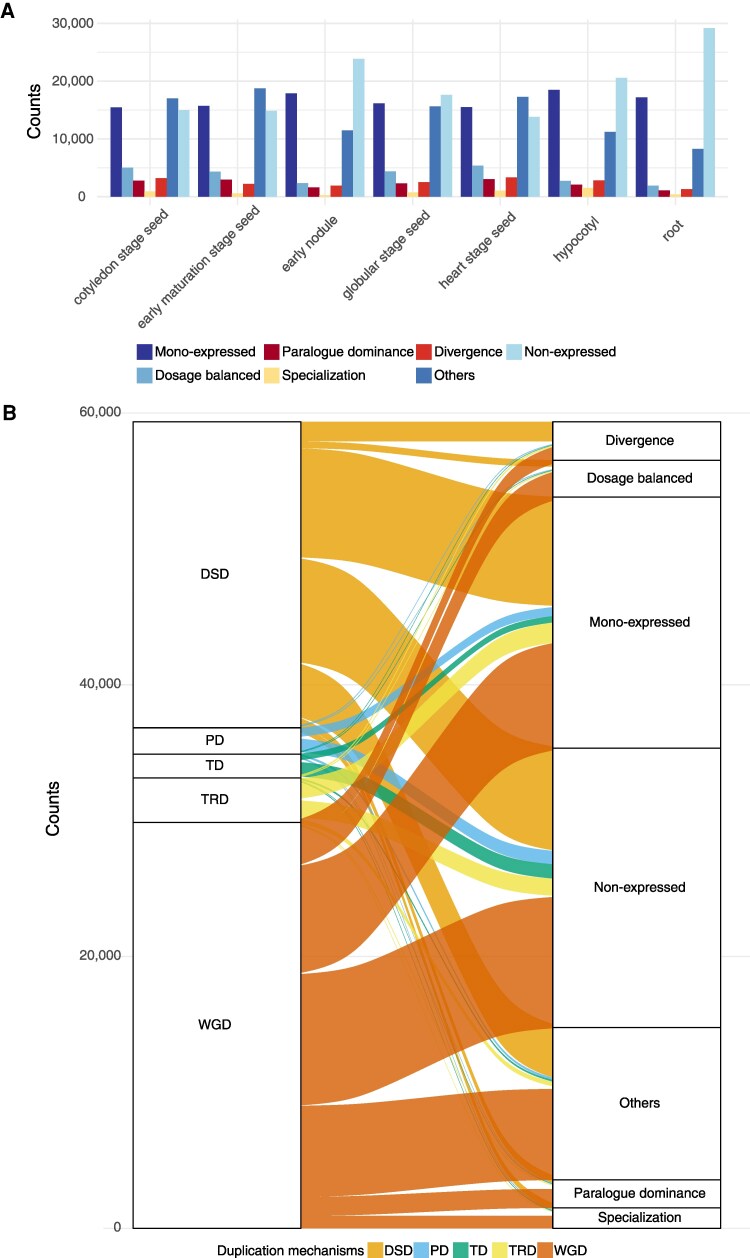
Distributions of distinct within-tissue expression patterns of duplicated gene pairs. **A)** Number of duplicated gene pairs classified by distinct expression patterns within a tissue, including mono-expressed, dosage balanced, paralogue dominance, specialization, divergence and others (as described in Fig. 1), as well as non-expressed category. **B)** Relationship between gene duplication mechanisms and within-tissue expression patterns. The left panel categorizes genes based on duplication mechanisms: whole-genome duplication (WGD), tandem duplication (TD), proximal duplication (PD), dispersed duplication (DSD), and transposed duplication (TRD). The right panel represents within-tissue gene expression patterns. Flow width indicates the relative frequency of each transition, highlighting how different duplication mechanisms contribute to within-tissue expression variation.

We then integrated the gene pair information with the single-cell expression profiles from each of the 7 tissues, root, hypocotyl, nodule, globular stage seed, heart stage seed, cotyledon stage seed, and early-maturation stage seed to examine the within-tissue expression dynamics (Zhang et al. 2025). Building on the methods developed by (Benoit et al. 2025), which measured expression correlation and differential expression of duplicated gene pairs across tissues, we adapted the approach to assess gene expression across cell types within tissue. In addition to the 4 previously described biologically meaningful expression patterns (Benoit et al. 2025)—(i) dosage balanced, where duplicates retain similar expression profiles and levels across cell types within a tissue (Fig. 1C); (ii) paralogue dominance, where duplicates share similar expression profiles but exhibit consistent level of differential expression across cell types (Fig. 1D); (iii) specialization, where duplicates display distinct expression profiles, exhibiting skewed differential expression in one or multiple cell types (Fig. 1E); (iv) divergence, where duplicates differ in both expression profiles and levels—we introduced a fifth category (Fig. 1F): (v) mono-expression, in which only one gene in the pair is expressed whereas the other remains inactive in a given tissue (Fig. 1G). Additionally, gene pairs in which neither gene is expressed in the examined tissues were classified as non-expression, whereas those that do not fit any of the above categories were grouped as “others” (Fig. 1H). Notably, specialization and divergence may represent the outcomes of subfunctionalization or neofunctionalization. Among the 5 biologically meaningful expression patterns, the mono-expression category consistently contained the most gene pairs within each individual tissue (Fig. 2A and Supplementary Table S3). This was followed by dosage balanced, divergence, paralogue dominance, and specialization patterns, except in the hypocotyl, where divergence gene pairs outnumbered dosage balanced ones, and in the early-maturation stage seed, where paralogue dominance exceeded divergence (Fig. 2A and Supplementary Table S3).

When examining the expression patterns of gene pairs grouped by their duplication mechanisms, we found that those derived from whole-genome duplication and dispersed duplication exhibited similar patterns, whereas gene pairs from tandem, proximal, and transposed duplication showed greater similarity to each other (Fig. 2B, Supplementary Fig. S1 and Supplementary Table S3). The unpredictable, seemingly random nature of dispersed duplication (Ganko et al. 2007; Qiao et al. 2019) may explain why the expression patterns of gene pairs derived from this mechanism differ from those derived from other small-scale duplication. Additionally, the mechanisms underlying the generation of dispersed duplication remain unclear with multiple possibilities proposed (Katju and Lynch 2003; Ganko et al. 2007). Given this uncertainty, we focused on gene pairs arising from tandem, proximal, and transposed duplication, collectively referred to as regional duplication due to their tendency to remain within specific chromosomal regions, to compare their expression patterns with those of gene pairs derived from whole-genome duplication. The proportion of expressed gene pairs, where at least one gene is active in a given tissue, varied by duplication mechanisms. For instance, in nodule tissue, about 60% of gene pairs from regional duplication (excluding transposed duplication) were not expressed, whereas only 37% of gene pairs derived from whole-genome duplication remained inactive (Supplementary Table S3). Among expressed regional duplicates, most exhibited mono-expression, with only a small fraction displaying other biologically meaningful expression patterns, such as dosage balanced, paralogue dominance, specialization, or divergence (Supplementary Table S3). These findings suggest that correlated expression patterns are more likely among whole-genome duplication derived than regional duplication derived, consistent with previous report in Arabidopsis, where duplicated genes originating from large-scale duplication and retained in duplicated segments exhibit more correlated expression patterns than those arising from small-scale duplication or those not located within duplicated segments (Casneuf et al. 2006; Qiao et al. 2019). These observations also support the gene balance hypothesis: whereas whole-genome duplication preserves the relative dosage of all interacting partners, small-scale duplication is more likely to disrupt this balance, leading to differential expression outcomes (Ohno 1970; Freeling 2009; Defoort et al. 2019; Kuzmin et al. 2022).

### Genetic variation in ACRs and its correlation with within-tissue expression patterns of duplicated gene pairs

*Cis*-regulatory variants are thought to drive transcriptional divergence in duplicated gene pairs and contribute to their retention (Iohannes and Jackson 2023); to explore this, we analyzed accessible chromatin regions (ACRs), where cis-regulatory elements reside, from scATAC-seq data in 7 tissues corresponding to the snRNA-seq data (Zhang et al. 2025). For each gene pair, we associated ACRs with duplicated genes based on their proximity.

**Figure 3.**
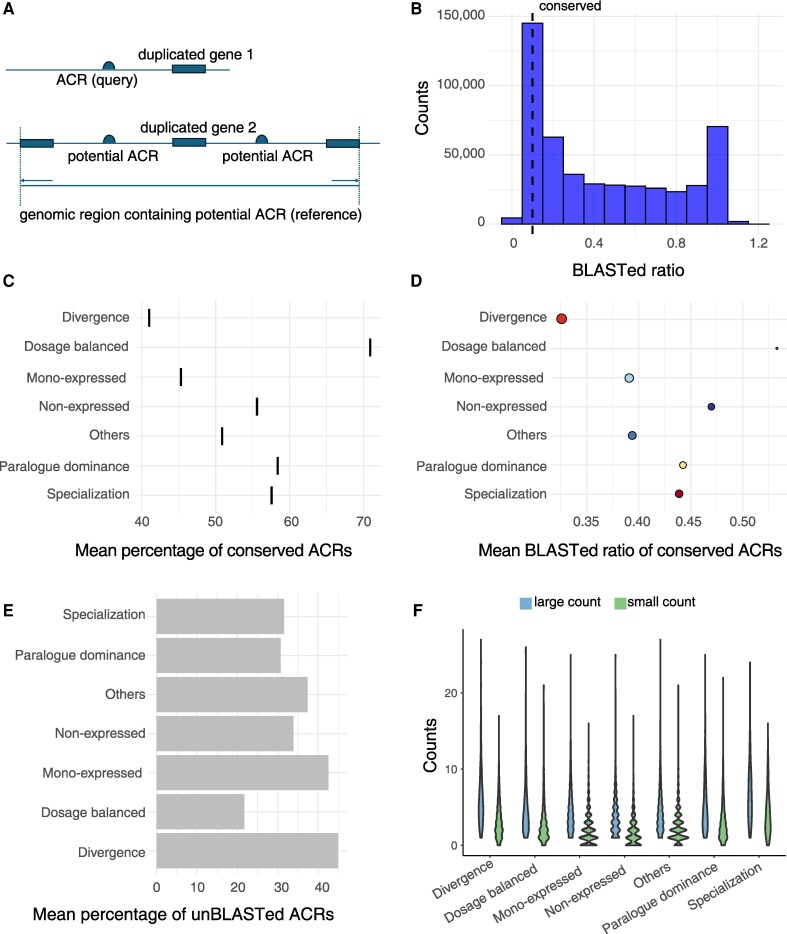
Genetic variation within ACRs associated with gene pairs with different within-tissue expression patterns. **A)** Illustration of the query and reference region for a BLAST: an ACR sequence associated with duplicated gene1 as a query, whereas the genomic region spanning from the upstream to the downstream gene of duplicated gene2 as a reference region. **B)** Number of ACRs BLASTed to the reference region in cotyledon stage seed. ACRs with a BLASTed ratio greater than 0.1 were classified as conserved. **C)** Mean percentage of conserved ACRs (black bars) across gene pairs with different within-tissue expression patterns in cotyledon stage seed. **D)** Mean BLASTed ratio of conserved ACRs across gene pairs with different within-tissue expression patterns in cotyledon stage seed. The size of dots represents the mean mismatch rate of conserved ACRs across gene pairs with different within-tissue expression patterns in cotyledon stage seed. The color of dots represents different expression patterns. **E)** Mean percentage of ACRs without a corresponding sequence in the reference region (unBLASTed ACRs) across gene pairs with different within-tissue expression patterns in cotyledon stage seed. **F)** Number of ACRs associated with each duplicated gene within gene pairs exhibiting different within-tissue expression patterns in cotyledon stage seed. Within each gene pair, the gene associated with a higher number of ACRs was classified into the large count group, whereas the one with fewer ACRs was classified into the small count group.

To assess genetic variation in ACRs associated with duplicated gene pairs, we used a BLAST-based approach following the method of (Mendieta et al. 2024). For each gene pair, the ACR sequence associated with one duplicated gene was used as the query, whereas the reference was defined as the genomic region spanning from the upstream to the downstream gene of the corresponding duplicate (Fig. 3A). This approach accounts for the possibility that the ACR linked to the corresponding duplicate may be positioned between it and either its upstream or downstream gene. Conserved ACRs were defined based on the BLASTed ratio, calculated as the length of the BLASTed sequence within the reference region divided by the length of the query ACR. ACRs with BLASTed ratios greater than 0.1, corresponding to approximately 50 bp in length, were considered conserved (Fig. 3B).

We found that gene pairs exhibiting dosage balanced had the highest percentage of conserved ACRs, whereas divergence gene pairs had the lowest (Fig. 3C, Supplementary Fig. S2A and Supplementary Table S4). Dosage balanced gene pairs exhibit the most similar expression profiles and levels (Fig. 1C), whereas divergence gene pairs show great divergence (Fig. 1F). We also tested stricter BLASTed ratio thresholds for defining conserved ACRs (>0.2, 0.4, 0.6, and 0.8). As expected, the proportion of conserved ACRs generally decreased with increasing thresholds (Supplementary Table S4). Nevertheless, the trend remained consistent: higher percentage of conserved ACR correlating with greater expression similarity between duplicated genes (Supplementary Table S4). Based on these observations, we focused subsequent analyses on conserved ACRs defined using the 0.1 threshold. Further examination of genetic variation between conserved ACRs and their corresponding BLASTed sequences, including BLASTed ratio and mismatch rate (calculated as the ratio of mismatches between the BLASTed sequence and the ACR query to the BLASTed sequence length), revealed greater ACR sequence divergence in gene pairs with divergence expression pattern (Fig. 3D, Supplementary Fig. S2B, and Supplementary Table S4). We also quantified unBLASTed ACRs, those lacking detectable counterparts in their associated duplicated genes, potentially resulting from de novo emergence or loss of cis-regulatory sequences. Divergence, “others” and mono-expressed gene pairs had relatively higher proportions of unBLASTed ACRs, particularly compared to dosage balanced gene pairs (Fig. 3E, Supplementary Fig. S2C and Supplementary Table S4).

Another interesting observation is that, when examining the number of ACRs associated with each gene within a pair, non-expressed and mono-expressed pairs more frequently exhibited asymmetric ACR association, where only one gene had linked ACRs whereas the other had none (Fig. 3F, Supplementary Fig. S2D, and Supplementary Table S5). In contrast, divergence gene pairs with even higher unBLASTed ratios did not exhibit this pattern (Fig. 3F, Supplementary Fig. S2D, and Supplementary Table S5). This suggests that while ACRs might be conserved at the sequence level, their chromatin accessibility can differ between duplicates, potentially influencing whether a gene is expressed or not. Together, these observations highlight a correlation between expression divergence and ACR dynamics, shaped both by sequence divergence between conserved ACRs and their corresponding counterparts, and by variation in accessibility despite sequence conservation.

When further examining genetic variation in ACRs associated with gene pairs grouped by different duplication mechanisms, distinct patterns emerged (Supplementary Fig. S3 and Supplementary Table S6). Tandem-duplicated gene pairs exhibited the highest proportion of conserved ACRs, the highest BLASTed ratios and the lowest mismatch rates (Supplementary Fig. S3), but this did not correspond to stronger expression correlation (e.g. dosage balanced or paralogue dominance) (Fig. 2B and Supplementary Fig. S1). This discrepancy may reflect limitations of our current method, which tends to map ACRs back to themselves for tandem-duplicated gene pairs (Fig. 3A), or it may result from other factors impacting transcriptional activities, such as positional effects, DNA folding or competition for transcription factors. In contrast, dispersed- and transposed-duplicated pairs displayed markedly different ACR genetic variation: low proportions of conserved ACRs, low BLASTed ratios, relatively high mismatch rates, and elevated fractions of unBLASTed ACRs (Supplementary Fig. S3 and Supplementary Table S6). Proximal-duplicated pairs, by comparison, exhibited variation patterns comparable to those of whole-genome duplication derived pairs, except with slightly higher BLASTed ratios (Supplementary Fig. S3 and Supplementary Table S6). These findings suggest that cis-regulatory elements are possibly more frequently co-duplicated with tandem and proximal duplicates, as they were with whole-genome duplicates in which cis-regulatory elements are entirely duplicated (Shoemaker et al. 2006), whereas variation in the presence or absence of duplicated cis-regulatory elements appears more common in transposed and disperse duplicates (Qiao et al. 2019). Collectively, these results indicate that different duplication mechanisms likely give rise to different types of cis*-*regulatory variants, thereby shaping transcriptional divergence differently.

### Transcriptional and ACR chromatin accessibility dynamics of duplicated gene pairs across tissues

**Figure 4.**
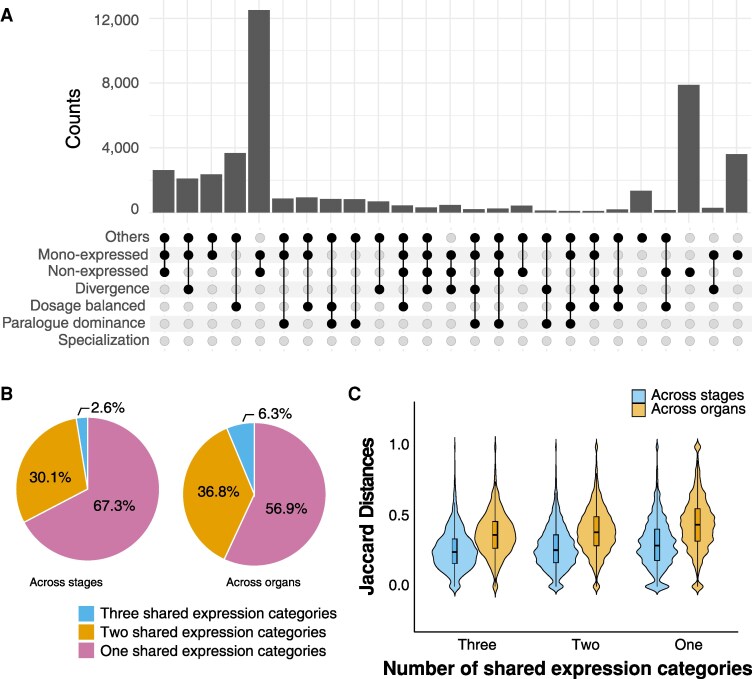
Across-tissue expression patterns of duplicated gene pairs. **A)** Number of duplicated gene pairs classified by combinations of expression patterns across 7 examined tissues. **B)** Pie charts showing the percentages of duplicated gene pairs with different number of shared expression categories across stages or across organs. **C)** Jaccard distance, which measures the overlap of ACR chromatin accessibility profiles across stages or organs, of duplicated gene pairs grouped by the number of shared expression categories across stages or across organs.

By assigning each gene pair an expression pattern—non-expressed, “others”, mono-expressed, dosage balanced, paralogue dominance, specialization and divergence—across the 7 examined tissues, we were able to characterize their cross-tissue transcriptional dynamics. In total, we identified 1,306 distinct expression combination patterns (Supplementary Table S7). Among the top 86 most prevalent patterns, each represented by at least 100 gene pairs, the most common involved gene pairs that were mono-expressed in at least one tissue and non-expressed in the remaining tissues, collectively accounting for over 20% of all gene pairs (Fig. 4A and Supplementary Table S7). Gene pairs with consistent mono-expression across all 7 tissues also ranked high, representing the third most common pattern (Fig. 4A and Supplementary Table S7). Notably, in these pairs, the same gene was always consistently expressed across tissues (Supplementary Fig. S4 and Supplementary Table S8), aligning with previous observations that differential expression within gene pairs tend to occur in the same direction across tissues (Roulin et al. 2013). The second most common pattern was complete non-expression across all tissues, which is unsurprising given the limited number of tissues included in this study (Fig. 4A and Supplementary Table S7).

The inclusion of multiple organs, root, hypocotyl, nodule, and seed, as well as seed sampled across distinct developmental stages, globular, heart, cotyledon, and early-maturation stages, further enabled us to assess the stability of gene pair expression patterns across both spatial and temporal dimensions. To facilitate interpretation, we consolidated expression patterns into 4 simplified categories: non-expressed, mono-expressed, correlated-expressed (dosage balanced, paralogue dominance, and specialization), and uncorrelated-expressed (divergence and “others”) (Supplementary Table S9). For each gene pair, we quantified the number of unique shared expression categories observed across organs or stages (See Methods for details). More than half of the gene pairs had a single shared expression category across stages (67.3%) and organs (56.9%) (Fig. 4B), indicating generally high consistency of expression patterns across tissues. Notably, 47% of gene pairs shared a single category across both stages and organs, although many of these were non- or mono-expressed (Supplementary Table S9). Fewer gene pairs shared a single category and more shared 2 or 3 categories across organs than across stages (Fig. 4B), suggesting greater transcriptional dynamics across organs. When examining expression stability of gene pairs grouped by their duplication mechanisms, these differences between across stages and across organs remained largely consistent; however, gene pairs derived from regional duplication exhibited a markedly higher proportion of only one shared category, compared with those derived from whole-genome or dispersed duplication (Supplementary Table S10 and Supplementary Fig. S5).

While gene pairs exhibit varied expression patterns across tissues, the genetic variants within their associated ACRs are determined by the underlying DNA sequence and remain unchanged across stages or organs. Consequently, these cross-tissue dynamics is unlikely to be driven directly by such genetic variants, aside from potential trans*-*effects but rather by whether the same or different ACRs are accessible across tissues. We quantified this variation using the Jaccard distance, which measures the overlap of ACR chromatin accessibility profiles across stages or organs, with higher values indicating less shared chromatin accessibility (Supplementary Tables S11 to S12). Surprisingly, the Jaccard distances showed little correlation with the number of shared expression categories across either stages or organs (Fig. 4C). Nevertheless, the higher Jaccard distances across organs than across stages (Fig. 4C), together with the greater expression dynamics observed across organs (Fig. 4B), suggests that variation in ACR chromatin accessibility profiles across tissues influences expression stability.

### Tracing the evolution of duplicated cis-regulatory regions following 2 rounds of whole-genome duplication

Soybean is a paleopolyploid species that has experienced 2 rounds of whole-genome duplication, theoretically giving rise to 4 duplicated genes sharing a single ancestral gene. Although extensive gene loss followed these duplications (Schmutz et al. 2010; Du et al. 2012; Zhao et al. 2017), the retained complete gene sets provide a valuable framework for investigating the evolution of cis-regulatory regions, duplicated alongside their associated genes (Li and Schmitz 2025), and its impact on gene expression. We focused on 4-gene sets, each derived from a single ancestral gene shared across all bean-like (papilionoid) legume species, identifying a total of 1,904 gene sets, comprising 7,616 genes (Supplementary Table S13). By leveraging phylogenetic trees of individual gene families across papilionoid species, we classified gene pairs as originating from either the recent or ancestral whole-genome duplication (Supplementary Table S13). Recently duplicated gene pairs exhibited higher similarity in protein sequences and expression profiles, compared with ancestrally duplicated ones (Supplementary Figs. S6 to S7 and Supplementary Tables S14 to S21). As expected, ACR sequences associated with recently duplicated genes pairs were also notably more similar than those associated with ancestrally duplicated pairs (Supplementary Fig. S8 and Supplementary Table S22 to S28).

**Figure 5.**
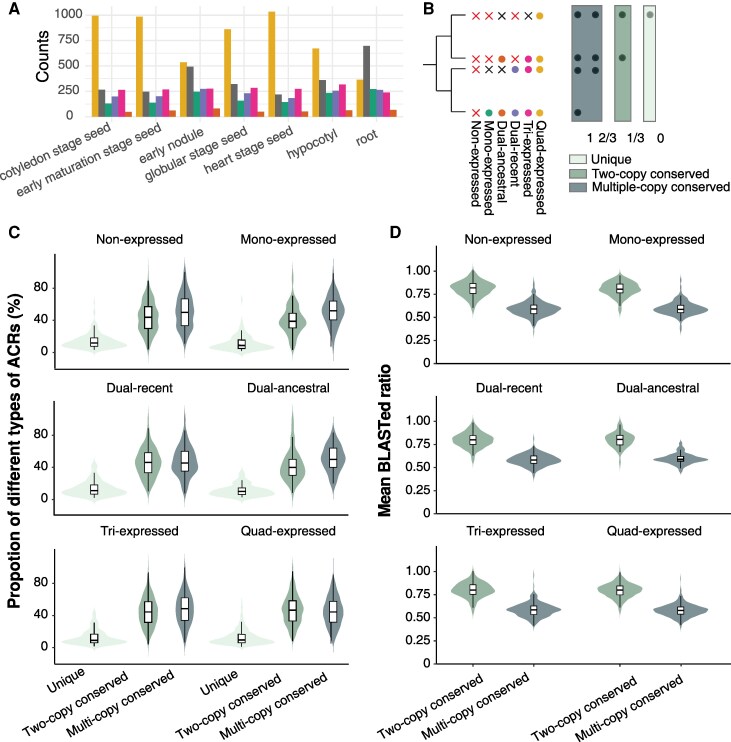
Proportions and sequence divergence of ACRs with different conservation states across gene sets with distinct expression patterns. **A)** Number of duplicated gene sets classified by the number of expressed genes, including, quad-expressed, tri-expressed, dual-recent, dual-ancestral, mono-expressed, and non-expressed. Different colors indicate distinct expression categories. **B)** Schematic of classifications of gene sets by the number of expressed genes and classification of ACRs based on their conservation count: unique ACR, 2-copy conserved ACR and multi-copy conserved ACR. Expressed genes are shown as dots (colored as in panel **A)**, and non-expressed genes as crosses (red if both recently duplicated genes are non-expressed, otherwise black). Three boxes indicate ACR conservation states, with color consistent across panels **B** to **D**. **C)** Proportions of ACRs with different conservation states associated with gene sets exhibiting different expression patterns in cotyledon stage seed. **D)** Mean BLASTed ratio of 2-copy conserved or multi-copy conserved ACRs across gene sets with different expression patterns in cotyledon stage seed.

Four-gene sets exhibited complex expression patterns, reflecting both the number of expressed genes and the varied expression correlations among them. To characterize this expression divergence, gene sets were first categorized by the number of expressed genes—non-, mono-, dual-, tri-, and quad-expressed, with dual expressed sets further subdivided into dual-recent (both expressed genes recently duplicated), and dual-ancestral (both ancestrally duplicated). Quad-expressed sets were the most frequent across most tissues, except in root, where non-expressed sets predominated (Fig. 5A and Supplementary Table S29). Dual-ancestral sets were consistently the least frequent, while other categories occurred at intermediate levels (Fig. 5A and Supplementary Table S29). Dual-recent sets always showed a strong skew toward high coexpression, reflecting their high expression similarity (Supplementary Fig. S9 and Supplementary Table S30). In contrast, other multi-expressed sets generally exhibited bimodal coexpression distributions, indicating either coordinated or divergent expression patterns, with the direction and magnitude of skew depending on both the number of expressed gene and the tissue context (Supplementary Fig. S9 and Supplementary Table S30).

To investigate the genetic variation and sequence origin of ACRs associated with these duplicated gene sets, we BLASTed each ACR associated with a duplicated gene against the reference regions of the other 3 duplicated genes within the same set. Each ACR was then assigned a conservation count, defined as the number of times it was conserved (BLASTed ratio > 0.1) across the 3 BLASTs. Based on this metric, ACRs were classified into 3 categories: unique ACRs, with no conserved sequences near any other duplicated genes, (conservation count = 0); 2-copy conserved ACRs, conserved between only 2 genes, most often the 2 genes derived from the most recent duplication (conservation count = 1); and multiple-copy conserved ACRs, conserved across 3 or 4 duplicated genes after 2 rounds of duplication (conservation count = 3 or 2, with the latter suggesting possible loss of ACR in one duplicate) (Fig. 5B).

The proportion of 2-copy or multi-copy conserved ACRs was generally similar, and substantially higher than the proportion of unique ACRs, a pattern consistent across gene sets with varying expression profiles (Fig. 5C, Supplementary Fig. S10 and Supplementary Table S31). These findings suggest that most ACRs associated with duplicated gene sets are conserved regions, likely retained from at least one round of whole-genome duplication, whereas only a small fraction are unique ACRs, potentially representing de novo regions that evolved at a low rate, although some unique ACRs may also result from loss in other duplicates. Notably, the relatively high frequency of multi-copy conserved ACRs was unexpected, given the extensive turnover of regulatory regions reported across multiple plant species with comparable divergence times (Lu et al. 2019). One possible explanation is that the divergence of non-coding sequences may be more pronounced across species than within a single-genome; however, this question requires further exploration in additional species and comparative contexts. We further calculated the average BLASTed ratio and mismatch rate for 2-copy and multi-copy conserved ACRs. The higher genetic variation observed in multi-copy compared with 2-copy conserved ACRs supports a temporal model of regulatory sequence divergence, in which ACRs gradually accumulate sequence variation over time (Fig. 5C, Supplementary Fig. S11 and Supplementary Table S31). Overall, however, there were no substantial differences in either proportion or genetic variation of these ACRs among gene sets with different expression patterns (Fig. 5, B and C, Supplementary Fig. S11 and Supplementary Table S31).

### Evolution of cell-type-specific expression and cell-type-specific ACRs

**Figure 6.**
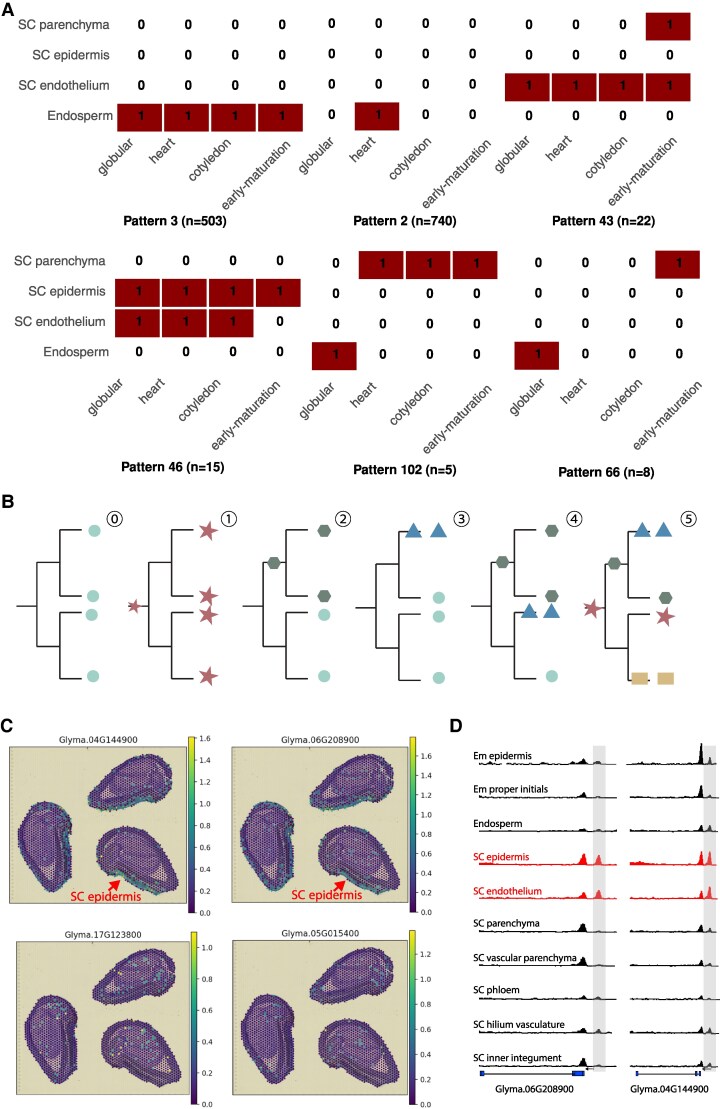
Evolution of cell-type-specific expression. **A)** Dynamics of cell-type-specific expression across stages, with ones indicating cell-type-specific expression in the given cell type and stage and zeros indicating absence of cell-type-specific expression. **B)** Schematic of initial expression ⓪ in duplicated gene sets and possible evolutionary trajectories of cell-type-specific expression: ① evolved before the first round of whole-genome duplication; ② evolved before the second round of whole-genome duplication; ③ evolved after the second round of whole-genome duplication; ④ and ⑤ combinations of scenarios. **C)** Spatial distribution of transcripts for a 4-gene set in cotyledon stage seed (cross-section), obtained from spatial RNA-Seq (https://soybean-atlas.com/). Dot color represents expression level, as indicated by the color scale. Gene IDs are labeled above each section. Two recently duplicated genes with seed-coat epidermic specific expression are highlighted with red arrows (top), while the other two do not show this pattern (bottom). **D)** The ACRs associated with the duplicated genes exhibiting seed-coat epidermic specific expression showing higher chromatin accessibility in this cell type and seed-coat endothelium. Em, embryo; SC, seed coat.

Genes with cell-type-specific expression are essential for establishing or maintaining cell identity, with cis-regulatory elements determining the cell types, developmental stages, and expression levels in which these genes are expressed (Wittkopp and Kalay 2012; Schmitz et al. 2022; Marand et al. 2023). We defined cell-type-specific genes as those showing significantly higher expression in a given cell type compared to an equal number of randomly sampled cells from other cell types within the same tissue (Zhang et al. 2025). On average, 8,278 cell-type-specific genes were identified per tissue, with heart stage seed (11,497) having the highest number and nodule (4,323) the lowest (Supplementary Table S32). Across developmental stages, these gene exhibited distinct dynamics: many maintained consistent cell-type-specific expression across stages (Fig. 6A—pattern 3), others were restricted to a single stage (Fig. 6A—pattern 2), and a smaller subset showed gains or losses of specificity during development (Fig. 6A—patterns 43 and 46), or shifts in specificity across stages (Fig. 6A—patterns 66 and pattern 102) (Supplementary Tables S33 to S34).

Placing these genes within the framework of 4-gene sets—where duplicates initially shared the same gene body and regulatory regions—allows us to trace the evolutionary trajectories of cell-type-specific expression and potentially uncover the regulatory regions driving these changes. We focused on the 4-gene sets in which all 4 genes were expressed and at least one exhibited cell-type-specific expression, thereby avoiding confounding effects from loss of expression in some duplicates. These duplicated genes were clustered using a binary matrix indicating the presence or absence of significant differential expression across cell types within the respective tissue (see Methods) (Supplementary Figs. S12 to S18). Based on the number of shared clusters within each set and the ratio of genes assigned to each cluster, we inferred distinct evolutionary paths of cell-type-specific expression (Fig. 6B) (Supplementary Table S35). A few sets were assigned to only a single cluster, suggesting that cell-type specificity likely originated before the first round of whole-genome duplication and was subsequently maintained through both rounds of duplication (Fig. 6B ①) (Supplementary Table S36). In contrast, most sets had multiple clusters, reflecting the divergence or emergence of novel expression patterns, which may have arisen before (Fig. 6B ②) or after the second round of whole-genome duplication (Fig. 6B ③); with some sets exhibiting combinations of these scenarios (Fig. 6B ④, ⑤) (Supplementary Table S36).

To explore the role of cis-regulatory activities in shaping these cell-type-specific expression dynamics within gene sets, we prioritized the analysis of their associated cell-type-specific ACRs (ctACRs) (Zhang et al. 2025). Specifically, we focused on ctACRs with significantly higher chromatin accessibility in the cell types where their associated duplicated genes were specifically expressed (see Methods) (Supplementary Table S37). Overall, conservation patterns of these ctACRs were broadly similar across gene sets with distinct evolutionary trajectories of cell-type-specific expression (Supplementary Fig. S19). Notably, gene sets forming a single cell-type-specific expression cluster tended to have a higher proportion of multi-copy conserved ctACRs compared to 2-copy conserved or unique ctACRs (Supplementary Fig. S19). These findings suggest that ctACRs with different conservation states may play distinct roles in the evolution of cell-type-specific expression.

Importantly, by integrating cell-type-specificity with regulatory sequence conservation, we were able to pinpoint candidate ACRs responsible for cell-type-specific expression. For example, in one 4-gene set, a recently duplicated pair displayed seed-coat epidermis specific expression in the cotyledon stage seed (Fig. 6C, top panel), whereas the other pair lacked cell-type-specific expression (Fig. 6C, bottom panel). Within this set, we identified 3 ACRs exhibiting significantly higher chromatin accessibility in seed-coat epidermis cells (Supplementary Table S37). Two of them also showed elevated chromatin accessibility in seed-coat endothelium and were each associated with one of the cell-type-specific genes separately (Fig. 6D). Additionally, these 2 ACRs showed relatively high sequence conservation (Supplementary Fig. S20). Together, these results suggest that the regulatory elements within these 2 ctACRs likely arose de novo after the first round of whole-genome duplication, were duplicated during the second duplication, and were ultimately retained, contributing to cell-type-specific expression in these 2 genes (Fig. 6C. top panel). Although experimental validation is still needed to confirm their functions, this approach demonstrates a powerful strategy for identifying regulatory regions that drive cell-type-specific expression and for advancing our understanding of the evolution of cell-type specificity. Moreover, the dataset generated here provides a valuable community resource for investigating specific cell types or duplicated gene sets of interest.

## Discussion

### Single-cell genomics: a key to understanding transcriptional divergence and genetic variants in chromatin accessibility

The transcriptional divergence of duplicated genes has been extensively studied in polyploid plants (Du et al. 2012; Roulin et al. 2013; Zhao et al. 2017; Bird et al. 2021; Wang et al. 2021; Han et al. 2022; Fang et al. 2023; Hu et al. 2024). However, most previous studies relied on bulk RNA-seq, assessing divergence primarily through differential expression at the tissue level, with only few extending analyses to the single-cell scale (Coate et al. 2020). Compared to bulk approaches, single-cell genomics revealed more nuanced forms of divergence, including shifts in expression across cell types within a tissue and altered expression patterns across tissues (Figs. 1 and 4). Characterizing both within- and across-tissue expression patterns provides a more comprehensive view of the spatiotemporal dynamics of gene expression. These multi-dimensional patterns also allow us to identify different drivers of expression divergence: genetic variation within ACRs primarily shapes within-tissue divergence, whereas variation in ACR chromatin accessibility profiles has a greater impact on cross-tissue divergence. These findings suggest that cis-regulatory elements probably act predominantly at the cellular level, and such patterns can best be captured through single-cell genomics.

Admittedly, bulk ATAC-seq, which profiles ACRs at the tissue level, remains powerful for detecting broad genome-wide patterns. In some cases, combining bulk ATAC-seq with snRNA-seq could identify some candidate regulatory regions underlying specific transcriptional patterns, for example, conserved ACRs with high BLASTed ratio and low mismatch rate associated with dosage balanced gene pairs or unBLASTed ACRs linked to mono- or non-expressed gene pairs. However, scATAC-seq provides cellular context for ACRs, which is particularly valuable for dissecting mechanisms of cell-type-specific expression. When integrated with snRNA-seq, scATAC can help identify candidate ctACRs responsible for conserved or shifted cell-type-specific expression among duplicated genes, as exemplified in our duplicated gene sets (Fig. 6, C and D). Nevertheless, pinpointing the causal regulatory elements within these ctACRs and understanding how these elements interact remains challenging.

### The evolution of cis-regulatory elements

The duplication of cis-regulatory elements represents a key source of novel regulatory sequences (McDonald and Reed 2024; Li and Schmitz 2025). Similar to protein-coding sequences of duplicated genes, which often experience reduced selective constraints (Kondrashov et al. 2002), duplicated cis-regulatory elements may also undergo relaxed selection, and accumulate mutations through genetic drift, potentially driving phenotypic novelty. This process is likely more pronounced following whole-genome duplication than small-scale duplication. Duplicates arising from whole-genome duplication tend to retain shared cis*-*regulatory elements (Shoemaker et al. 2006), whereas those from small-scale duplication may not (Arsovski et al. 2015). Consistent with this, we observed a higher percentage of ACRs that can be BLASTed to corresponding duplicated regions in whole-genome duplication derived gene pairs compared to small-scale duplication derived gene pairs, particularly transposed and dispersed duplicates (Supplementary Fig. S4).

On other hand, transposed or dispersed duplicated genes, where the gene body and likely its associated regulatory elements are translocated to new genomic environments, may come under the influence of regulatory elements pre-existed in their new surroundings. Alternatively, de novo regulatory elements could arise from sequences that were previously non-regulatory (Katikaneni and Lowe 2025; Li and Schmitz 2025). Both processes likely contribute to the greater divergence expression patterns observed in these duplicates. Together, modifications of duplicated elements and the de novo emergence of regulatory elements increase the diversity of regulatory sequences. Moreover, our observation that most ACRs had its duplicated counterparts remained in the genome, with only a small subset of ACRs evolving de novo, suggest that retention and modification of existing elements might occur more frequently than de novo emergence.

Nevertheless, the precise genetic mechanisms and cis-regulatory activities that drive distinct expression outcomes—such as paralogue dominance versus specialization—remain unresolved. Still, other mechanisms, including epigenetic modifications such as DNA methylation and histone modifications, can also attribute to expression divergence. On the other hand, the relative contributions of evolutionary forces, including drift and selection, in shaping variation within existing cis-regulatory elements or in facilitating the emergence of novel ones are not yet fully understood. Finally, the interplay between the evolution of cis-regulatory elements and their associated protein-coding sequence remains underexplored.

### Cell-type-specific expression in the framework of duplicated genes

The 4-gene sets involved in cell-type-specific expression used in this study provide a powerful framework for understanding the evolution of cell-type-specific expression. The diverse evolutionary trajectories of cell-type-specific expression were systematically explored and classified (Fig. 6B). Practically, this framework also enables the identification of candidate ACRs underlying cell-type-specific expression, as exemplified by seed-coat epidermal specific gene sets (Fig. 6, C and D). Identifying these regulatory sequences not only advances our understanding of cell identity formation but also has potential application in synthetic biology, for instance, in designing regulatory regions to drive expression in specific cell types.

Furthermore, these dynamics of cell-type-specific expression provide a valuable perspective for studying subfunctionalization and neofunctionalization, the canonical outcomes of duplicated genes over long evolutionary time (Ohno 1970; Lynch and Conery 2000; Birchler and Yang 2022; Kuzmin et al. 2022; Gout et al. 2023). A persistent challenge has been defining or quantifying these processes, but changes in gene expression, particularly cell-type-specific changes, can serve as informative indicator. For example, Tran et al. (2024) demonstrated that reduced expression of one duplicated BLADE-ON-PETIOLE (BOP) gene in the bract-initiating region of *Capsella rubella* impaired its ability to regulate floral organ number and suppress bract formation, representing a step toward subfunctionalization. Building on this concept, our examination of cell-type-specific expression dynamics in duplicated genes provides a more quantitative means of evaluating these evolutionary outcomes. Integrating single-cell genomics with the comparative studies, essential for inferring ancestral expression patterns, offers a promising path to dissect subfunctionalization and neofunctionalization in greater detail.

In summary, understanding the evolution of paralogs is crucial, as many crops, including Solanum, grasses, and legumes, have similarly complex gene families that undoubtedly influence agriculturally important traits. Gaining insights into the transcriptional divergence of duplicated genes and the underlying cis-regulatory dynamics can inform strategies for fine-tuning gene dosage, facilitating the engineering of quantitative variation, and overcoming redundancy barriers in functional studies.

## Materials and methods

### Duplicated gene pairs and gene sets in soybean

We identified duplicated gene pairs in soybean (*Glycine max*) using the DupGen_finder pipeline (https://github.com/qiao-xin/DupGen_finder), with common bean (*Phaseolus vulgaris*) as the outgroup reference. We first performed all-against-all BLASTp within the soybean genome and all-to-all BLASTp between the soybean and *P. vulgaris* genomes, using the parameters “–sensitive –max-target-seqs 5 –evalue 1e-10”. The resulting BLASTed results were further filtered to retain only hits with sequence identity > 40% and coverage >70%. To retain redundancy across duplication modes, we used DupGen_finder.pl script, which classified duplicated gene pairs into 5 categories: whole-genome duplication, tandem duplication, proximal duplication, transposed duplication, and dispersed duplication (Fig. 1). Gene pairs located in scaffolds were excluded from further analyses.

To identify gene sets consisting exclusively of 4 whole-genome duplication derived genes, presumably originating from 2 rounds of whole-genome duplication in soybean, we used the legume gene family dataset from SoyBase (https://www.soybase.org/), specifically the file “legume.fam3.VLMQ.sup1A_hsh.tsv”, which contains gene ID information for gene families shared across all papilionoid legume species and the corresponding phylogenetic trees (“legume.fam3.VLMQ.sup1B_trees”) built for each individual family. We first filtered the dataset to retain only gene families containing exactly 4 duplicated genes in soybean, and then further refined by cross-referencing with our previously identified whole-genome duplication derived gene pairs to ensure all 4 genes were products of whole-genome duplication. Finally, we used a customized script to identify the gene pairs clustered within the same clade of gene tree, suggesting they likely arose from recent whole-genome duplication.

### Expression dynamics of gene pairs or gene sets

Raw gene counts from 7 tissues—root, hypocotyl, nodule, globular stage seed, heart stage seed, cotyledon stage seed, and early-maturation stage seed—were obtained from snRNA-seq (Zhang et al. 2025). Data from each tissue were analyzed separately. Only genes with 2 replicates per cell type within the same tissue were retained for downstream analyses. For each tissue, we calculated the Spearman correlation between replicates of cell type and excluded cell types with low correlation (0.75 or blow). To ensure consistency between cell types identified in the snRNA-seq and scATAC-seq datasets, we focused only on cell types shared by both. Lowly expressed genes were filtered using “filterByExpr” function, and the remaining gene counts were normalized using “cpm” function in edgeR (Chen et al. 2025). Genes with zero expression across all cell types within a tissue were classified as “non-expressed” and excluded from further analysis. The remaining genes were used to construct coexpression networks (Benoit et al. 2025) to assess whether duplicated gene pairs exhibit similar expression profiles across cell types within a tissue.

To quantify expression relationships between duplicated genes within the same pair, we calculated the absolute mean and SD of log2-transformed fold change across cell types within the same tissue (Benoit et al. 2025). Following Benoit et al. (2025), we classified duplicated gene pairs into 6 categories based on coexpression and variation in expression levels, introducing “mono-expression” as an additional category:

1. Dosage balanced: coexpression > 0.9, mean log_2_ fold change <1, SD log_2_ fold change <1
2. Paralogue dominance: coexpression > 0.9, mean log_2_ fold change ≥ 1, SD log_2_ fold change <1
3. Specialization: coexpression > 0.9, mean log_2_ fold change ≥ 1, SD log_2_ fold change ≥1
4. Divergence: coexpression < 0.5, mean log_2_ fold change ≥ 1, SD log_2_ fold change ≥1
5. Mono-expression: coexpression = NA.
6. Others: expressed gene pairs that did not fall into any of the above categories.

To investigate the dynamics of these expression patterns across tissues—particularly across developmental stages and different organs—we consolidated them into 4 simplified categories: non-expressed, mono-expressed, correlated-expressed (including dosage balance, paralogue dominance, and specialization), and uncorrelated-expressed (including divergence and “others”). We then assessed the number of shared expression categories across seeds collected at 4 developmental stages (globular, heart, cotyledon, and early-maturation) and across 4 organs (hypocotyl, root, nodule, and heart stage seed). Non-expression was treated as a distinct case: it was excluded when evaluating shared expression categories if co-occurring with any expressed category and was only assigned when a gene pair was non-expressed across all organs or stages.

To assess transcriptional divergence of 4-gene sets, we treated the 4 genes as 6 gene pairs: 2 representing recently duplicated pairs (each clustered into separate clades in the phylogenetic trees of the respective gene families) and 4 representing ancestrally duplicated pairs (Supplementary Table S13). For each gene pair, we calculated coexpression values, the absolute mean and SD of log2-transformed fold change, following the same approach used above to examine expression divergence.

### Identification of cell-type-specific genes in each tissue

Cell-type-specific genes were identified using differential expression analysis for each cell type within its respective tissue (Zhang et al. 2025). Gene counts were aggregated across nuclei of the same cell type and compared to gene counts of an equal number of randomly sampled cells from other cell types within the same tissue, using edgeR (Chen et al. 2025). Genes with a log_2_ fold change > 1 and an adjusted *P*-value < 0.05 were classified as highly expressed in a given cell type. A gene was defined as cell-type specific if it was classified as highly expressed in at least one cell type within its tissue. To further characterize their dynamics across tissues, we examined cell-type-specificity across the same cell types—endosperm, seed-coat endothelium, seed-coat epidermis, and seed-coat parenchyma—in seeds sampled at 4 different developmental stages (globular, heart, cotyledon, and early-maturation).

To investigate the evolution of cell-type-specific expression among gene sets, we first filtered for gene sets in which 4 duplicated genes were expressed and at least one duplicate exhibited cell-type-specific expression. For these genes, we constructed a binary matrix representing the presence (1) or absence (0) of significant differential expression across cell types within the respective tissue. Rows with no variation across cell types (i.e. all 0 s) were removed prior to clustering. Pairwise distances between genes were calculated using the Jaccard distance, defined as 1 minus the ratio of shared presences to total presences and absences between 2 genes. Agglomerative hierarchical clustering was performed using the Ward.D2 linkage method. The optimal number of clusters was evaluated using the elbow method (within-cluster sum of squares) and silhouette analyses (average silhouette width) based on hierarchical clustering with Ward’D2 method. Genes were then assigned to clusters by cutting the dendrogram at the selected value of k. The resulting clusters observed within the gene set reflect the potentially varied evolutionary trajectories of cell-type-specific expression. When all 4 genes grouped into a single cluster, cell-type-specific expression was inferred to have evolved prior to the first whole-genome duplication (Fig. 6B ①). A 2-cluster distribution with a 2:2 ratio indicated evolution prior to the second whole-genome duplication (Fig. 6B ②), whereas a 1:3 ratio suggested evolution after the second whole-genome duplication (Fig. 6B ③). A 3-cluster distribution with a 1:2:1 ratio was interpreted as a combination of these scenarios (Fig. 6B ④). Finally, when 4 clusters with a 1:1:1:1 ratio were observed, cell-type-specific expression was considered to reflect a more complex integration of the above evolutionary processes (Fig. 6B ⑤).

### Genetic variation in ACRs associated with gene pairs or gene sets

ACRs identified in soybean was obtained from Zhang et al. (2025) . The gene closest to each ACR was defined as the gene-associated ACR, and this association was determined using bedtools (Quinlan and Hall 2010). BLAST analysis was performed to examine the sequence divergence of ACRs associated with the 2 genes within the same duplication gene pair. The sequence of the ACR associated with one duplicated gene was used as the query, while the sequence of the 2 adjacent genes from the other duplicated gene served as the reference (Fig. 3A). These sequences were isolated using the “getfasta” function in bedtools (Quinlan and Hall 2010). Then sequence comparisons were performed using BLASTn with the following parameters “-task blastn-short -evalue 1e-3 -max_target_seqs 4-word_size 7 -gapopen 5 -gapextend 2 -penalty −1 -reward 1 -dust no -outfmt 6” following (Mendieta et al. 2024).

ACRs were classified as conserved if more than 10% (approximately 50 bp) were BLASTed to the reference region. The mean percentage of conserved ACRs were calculated for each gene pair by dividing the number of conserved ACRs by the total number of ACRs associated with that pair and then averaged across gene pairs with the same expression pattern. The BLASTed ratio was calculated by dividing the length of the BLASTed sequence in the reference region by the length of the query ACR. The mismatch rate was calculated as the number of mismatches between the ACR and the BLASTed sequence, divided by the length of the BLASTed sequence in the reference region. The percentage of unBLASTed ACRs was calculated by dividing the number of ACRs could not be BLASTed to the reference region by the total number of ACRs associated with the gene pair. The average BLASTed ratio, mismatch rate, and percentage of unBLASTed ACRs across all the ACRs associated with the gene pair were calculated to represent the level of sequence divergence of ACRs for each gene pair.

When measuring ACR sequence divergence across duplicated genes with the gene sets, we classified the ACRs based on conservation count, defined as the number of times an ACR was conserved (with a BLASTed ratio > 0.1) across the 3 BLASTs. Based on this metric, ACRs were categorized as unique ACRs, 2-copy conserved ACRs, and multi-copy conserved ACRs (Fig. 5B). We examined the compositions of these ACRs for ACRs associated with each gene set individually. For each gene set, we calculated the average BLASTed ratio and mismatch rate for 2-copy conserved ACRs, and multiple-copy conserved ACRs associated with each gene set. When comparing the ACRs explaining cell-type-specific expression, we specifically focused on cell-type-specific ACRs exhibiting higher chromatin accessibility in the cell types where their associated genes were specifically expressed (Zhang et al. 2025).

Jaccard distance was calculated as 1 minus the ratio of shared presences to total presences among all ACRs associated with each gene set across developmental stages (globular, heart, cotyledon, and early-maturation stage seed) or organs (hypocotyl, root, early nodule, and heart stage seed), providing a measure of the overlap in ACR accessibility profiles, where higher values indicate lower overlap (i.e. less shared chromatin accessibility).

All scripts for data processing and analysis are available at the following GitHub repository: https://github.com/Xianglichina/soybean_duplicated_genes_single_cell.git. During the preparation of this manuscript, we used ChatGPT to refine code and improve readability and language. All content was subsequently reviewed and edited by the authors, who take full responsibility for the final version.

## Supplementary Material

koaf279_Supplementary_Data

## Data Availability

All data are incorporated into the article and its online supplementary material.

## Supplementary data

The following materials are available in the online version of this article.

**Supplementary Figure S1.** Relationship between duplication mechanisms and within-tissue expression patterns of duplicated gene pairs across different tissues.

**Supplementary Figure S2.** Genetic variation within ACRs associated with gene pairs with different within-tissue expression patterns across different tissues.

**Supplementary Figure S3.** Genetic variation within ACRs associated with gene pairs derived from different duplication mechanisms.

**Supplementary Figure S4.** Histogram showing counts of mono-expressed gene pairs, comparing whether the same or different genes are expressed across tissues.

**Supplementary Figure S5.** Pie charts of gene pairs from different duplication mechanisms, with each chart showing the percentages of duplicated gene pairs grouped by the number of shared expression categories across developmental stages or organs.

**Supplementary Figure S6.** Comparison of protein sequence similarity between ancestrally and recently duplicated gene pairs.

**Supplementary Figure S7.** Comparison of expression divergence between ancestrally and recently duplicated gene pairs within each individual tissue.

**Supplementary Figure S8.** Comparison of sequence divergence of ACRs associated with ancestrally and recently duplicated gene pairs within each individual tissue.

**Supplementary Figure S9.** Transcriptional divergence of four-gene sets within each individual tissue measured both by the number of expressed genes per set and their expression correlations.

**Supplementary Figure S10.** Percentages of each ACR conservation type identified in four-gene sets exhibiting different expression patterns within each individual tissue.

**Supplementary Figure S11.** Sequence similarity of two-copy conserved versus multi-copy conserved ACRs associated with four-gene sets exhibiting distinct expression patterns within each individual tissue.

**Supplementary Figure S12.** Heatmap of the clusters identified across four gene sets, each containing at least one gene with cell-type-specific expression in cotyledon stage seed.

**Supplementary Figure S13.** Heatmap of the clusters identified across four gene sets, each containing at least one gene with cell-type-specific expression in early maturation stage seed.

**Supplementary Figure S14.** Heatmap of the clusters identified across four gene sets, each containing at least one gene with cell-type-specific expression in nodule.

**Supplementary Figure S15.** Heatmap of the clusters identified across four gene sets, each containing at least one gene with cell-type-specific expression in globular stage seed.

**Supplementary Figure S16.** Heatmap of the clusters identified across four gene sets, each containing at least one gene with cell-type-specific expression in heart stage seed.

**Supplementary Figure S17.** Heatmap of the clusters identified across four gene sets, each containing at least one gene with cell-type-specific expression in hypocotyl.

**Supplementary Figure S18.** Heatmap of the clusters identified across four gene sets, each containing at least one gene with cell-type-specific expression in root.

**Supplementary Figure S19.** Percentages of each ACR conservation type identified in cell-type-specific ACRs associated with four-gene sets exhibiting different evolutionary trajectories of cell-type-specific expression.

**Supplementary Figure S20.** Sequence alignment of the two ctACRs associated with recently duplicated gene pairs that exhibit seed coat epidermis–specific expression patterns (as shown in Fig. 6C).

**Supplementary Table S1.** Duplicated gene pairs identified in soybean.

**Supplementary Table S2.** Percentage of duplicated gene pairs derived from different duplication mechanisms in soybean.

**Supplementary Table S3.** Number and percentage of gene pairs exhibiting different within-tissue expression patterns in each tissue.

**Supplementary Table S4.** Characterization of genetic variation in ACRs associated with gene pairs exhibiting different within-tissue expression patterns in each tissue.

**Supplementary Table S5.** Percentage of gene pairs with both genes associated with ACRs or only one gene associated with ACRs for each within-tissue expression pattern in each tissue.

**Supplementary Table S6.** Characterization of genetic variation in ACRs associated with gene pairs grouped by different duplication mechanisms in each tissue.

**Supplementary Table S7.** Combinations of expression patterns across seven tissues and their frequencies.

**Supplementary Table S8.** Expression patterns of duplicated gene pairs across seven tissues.

**Supplementary Table S9.** Simplified expression categories for each gene pair across seven tissues and number of shared expression categories across stages and organs.

**Supplementary Table S10.** Counts and percentages for gene pairs (separated by different duplication mechanisms) with different number of shared expression categories across stages or across organs.

**Supplementary Table S11.** Jaccard distances for gene pairs across stages.

**Supplementary Table S12.** Jaccard distances for gene pairs across organs.

**Supplementary Table S13.** Gene pairs identified in each four-gene set, with the annotation of recently duplicated or ancestrally duplicated gene pairs.

**Supplementary Table S14.** Average percentage identity and mismatches of protein sequence of gene pairs within each four-gene set.

**Supplementary Table S15.** Characterization of expression patterns in cotyledon-stage seed tissue for gene pairs within each set.

**Supplementary Table S16.** Characterization of expression patterns in early maturation-stage seed tissue for gene pairs within each set.

**Supplementary Table S17.** Characterization of expression patterns in nodule tissue for gene pairs within each set.

**Supplementary Table S18.** Characterization of expression patterns in globular-stage seed tissue for gene pairs within each set.

**Supplementary Table S19.** Characterization of expression patterns in heart-stage seed tissue for gene pairs within each set.

**Supplementary Table S20.** Characterization of expression patterns in hypocotyl tissue for gene pairs within each set.

**Supplementary Table S21.** Characterization of expression patterns in root tissue for gene pairs within each set.

**Supplementary Table S22.** ACRs associated with gene sets in cotyledon stage seed.

**Supplementary Table S23.** ACRs associated with gene sets in early-maturation stage seed.

**Supplementary Table S24.** ACRs associated with gene sets in nodule.

**Supplementary Table S25.** ACRs associated with gene sets in globular stage seed.

**Supplementary Table S26.** ACRs associated with gene sets in heart stage seed.

**Supplementary Table S27.** ACRs associated with gene sets in hypocotyl.

**Supplementary Table S28.** ACRs associated with gene sets in root.

**Supplementary Table S29.** Counts of gene sets with distinct expression patterns within each tissue.

**Supplementary Table S30.** Characterization of expression patterns for gene sets in each tissue.

**Supplementary Table S31.** Characterization of different conservation-type ACRs associated with each gene set in each tissue.

**Supplementary Table S32.** Cell-type-specific genes identified in each tissue with matrix indicating the cell type(s) with significantly differential expression.

**Supplementary Table S33.** Cell-type-specific expression across stages.

**Supplementary Table S34.** Patterns of cell-type-specific expression across stages and their frequences.

**Supplementary Table S35.** Shared clusters and their corresponding counts within each four-gene set in each tissue.

**Supplementary Table S36.** Number of four-gene sets with different evolutionary trajectories of cell-type-specific expression.

**Supplementary Table S37.** Cell-type-specific ACRs exhibiting significantly higher chromatin accessibility in the same cell types where their associated duplicated genes expressed in individual tissue.

## Dive Curated Terms

The following phenotypic, genotypic, and functional terms are of significance to the work described in this paper:

## References

[koaf279-B1] Arsovski AA, Pradinuk J, Guo XQ, Wang S, Adams KL. Evolution of cis-regulatory elements and regulatory networks in duplicated genes of Arabidopsis. Plant Physiol. 2015:169(4):2982–2991. 10.1104/pp.15.00717 26474639 PMC4677880

[koaf279-B2] Benoit M, Jenike KM, Satterlee JW, Ramakrishnan S, Gentile I, Hendelman A, Passalacqua MJ, Suresh H, Shohat H, Robitaille GM, et al Solanum pan-genetics reveals paralogues as contingencies in crop engineering. Nature. 2025:640(8057):135–145. 10.1038/s41586-025-08619-6 40044854 PMC11964936

[koaf279-B3] Birchler JA, Yang H. The multiple fates of gene duplications: deletion, hypofunctionalization, subfunctionalization, neofunctionalization, dosage balance constraints, and neutral variation. Plant Cell. 2022:34(7):2466–2474. 10.1093/plcell/koac076 35253876 PMC9252495

[koaf279-B4] Bird KA, Niederhuth CE, Ou S, Gehan M, Pires JC, Xiong Z, VanBuren R, Edger PP. Replaying the evolutionary tape to investigate subgenome dominance in allopolyploid Brassica napus. New Phytol. 2021:230(1):354–371. 10.1111/nph.17137 33280122 PMC7986222

[koaf279-B5] Blanc G, Wolfe KH. Widespread paleopolyploidy in model plant species inferred from age distributions of duplicate genes. Plant Cell. 2004:16(7):1667–1678. 10.1105/tpc.021345 15208399 PMC514152

[koaf279-B6] Cai H, Des Marais DL. Expression variability following gene duplication facilitates gene retention. bioRxiv 2024:2024.11.06.622370. 10.1101/2024.11.06.622370, 7 November 2024, preprint: not peer reviewed.

[koaf279-B7] Casneuf T, De Bodt S, Raes J, Maere S, Van de Peer Y. Nonrandom divergence of gene expression following gene and genome duplications in the flowering plant Arabidopsis thaliana. Genome Biol. 2006:7(2):R13. 10.1186/gb-2006-7-2-r13 16507168 PMC1431724

[koaf279-B8] Chaudhary B, Flagel L, Stupar RM, Udall JA, Verma N, Springer NM, Wendel JF. Reciprocal silencing, transcriptional bias and functional divergence of homeologs in polyploid cotton (gossypium). Genetics. 2009:182(2):503–517. 10.1534/genetics.109.102608 19363125 PMC2691759

[koaf279-B9] Chen Y, Chen L, Lun ATL, Baldoni PL, Smyth GK. Edger v4: powerful differential analysis of sequencing data with expanded functionality and improved support for small counts and larger datasets. Nucleic Acids Res. 2025:53(2):gkaf018. 10.1093/nar/gkaf018 39844453 PMC11754124

[koaf279-B10] Coate JE, Farmer AD, Schiefelbein JW, Doyle JJ. Expression partitioning of duplicate genes at single cell resolution in Arabidopsis roots. Front Genet. 2020:11:596150. 10.3389/fgene.2020.596150 33240334 PMC7670048

[koaf279-B11] Defoort J, Van de Peer Y, Carretero-Paulet L. The evolution of gene duplicates in angiosperms and the impact of protein-protein interactions and the mechanism of duplication. Genome Biol Evol. 2019:11(8):2292–2305. 10.1093/gbe/evz156 31364708 PMC6735927

[koaf279-B12] Du J, Tian Z, Sui Y, Zhao M, Song Q, Cannon SB, Cregan P, Ma J. Pericentromeric effects shape the patterns of divergence, retention, and expression of duplicated genes in the paleopolyploid soybean. Plant Cell. 2012:24(1):21–32. 10.1105/tpc.111.092759 22227891 PMC3289580

[koaf279-B13] Fang C, Jiang N, Teresi SJ, Platts AE, Agarwal G, Niederhuth C, Edger PP, Jiang J. Dynamics of accessible chromatin regions and subgenome dominance in octoploid strawberry. Nat Commun. 2024:15(1):1–14. 10.1038/s41467-023-43650-z 38509076 PMC10954716

[koaf279-B14] Fang C, Yang M, Tang Y, Zhang L, Zhao H, Ni H, Chen Q, Meng F, Jiang J. Dynamics of *cis*-regulatory sequences and transcriptional divergence of duplicated genes in soybean. Proc Natl Acad Sci U S A. 2023:120(44):e2303836120. 10.1073/pnas.2303836120 37871213 PMC10622917

[koaf279-B15] Freeling M . Bias in plant gene content following different sorts of duplication: tandem, whole-genome, segmental, or by transposition. Annu Rev Plant Biol. 2009:60(1):433–453. 10.1146/annurev.arplant.043008.092122 19575588

[koaf279-B16] Ganko EW, Meyers BC, Vision TJ. Divergence in expression between duplicated genes in Arabidopsis. Mol Biol Evol. 2007:24(10):2298–2309. 10.1093/molbev/msm158 17670808

[koaf279-B17] Gout J-F, Hao Y, Johri P, Arnaiz O, Doak TG, Bhullar S, Couloux A, Guérin F, Malinsky S, Potekhin A, et al Dynamics of gene loss following ancient whole-genome duplication in the cryptic Paramecium complex. Mol Biol Evol. 2023:40(5):msad107. 10.1093/molbev/msad107 37154524 PMC10195154

[koaf279-B18] Haberer G, Hindemitt T, Meyers BC, Mayer KFX. Transcriptional similarities, dissimilarities, and conservation of cis-elements in duplicated genes of Arabidopsis. Plant Physiol. 2004:136(2):3009–3022. 10.1104/pp.104.046466 15489284 PMC523363

[koaf279-B19] Han J, Lopez-Arredondo D, Yu G, Wang Y, Wang B, Wall SB, Zhang X, Fang H, Barragán-Rosillo AC, Pan X, et al Genome-wide chromatin accessibility analysis unveils open chromatin convergent evolution during polyploidization in cotton. Proc Natl Acad Sci U S A. 2022:119(44):e2209743119. 10.1073/pnas.2209743119 36279429 PMC9636936

[koaf279-B20] Hu G, Grover CE, Vera DL, Lung P-Y, Girimurugan SB, Miller ER, Conover JL, Ou S, Xiong X, Zhu D, et al Evolutionary dynamics of chromatin structure and duplicate gene expression in diploid and allopolyploid cotton. Mol Biol Evol. 2024:41(5):msae095. 10.1093/molbev/msae095 38758089 PMC11140268

[koaf279-B21] Innan H, Kondrashov F. The evolution of gene duplications: classifying and distinguishing between models. Nat Rev Genet. 2010:11(2):97–108. 10.1038/nrg2689 20051986

[koaf279-B22] Iohannes SD, Jackson D. Tackling redundancy: genetic mechanisms underlying paralog compensation in plants. New Phytol. 2023:240(4):1381–1389. 10.1111/nph.19267 37724752

[koaf279-B23] Katikaneni A, Lowe CB. Novelty versus innovation of gene regulatory elements in human evolution and disease. Curr Opin Genet Dev. 2025:90:102279. 10.1016/j.gde.2024.102279 39591813 PMC11769741

[koaf279-B24] Katju V, Lynch M. The structure and early evolution of recently arisen gene duplicates in the Caenorhabditis elegans genome. Genetics. 2003:165(4):1793–1803. 10.1093/genetics/165.4.1793 14704166 PMC1462873

[koaf279-B25] Koenen EJM, Ojeda DI, Bakker FT, Wieringa JJ, Kidner C, Hardy OJ, Pennington RT, Herendeen PS, Bruneau A, Hughes CE. The origin of the legumes is a complex paleopolyploid phylogenomic tangle closely associated with the Cretaceous-Paleogene (K-Pg) mass extinction event. Syst Biol. 2021:70(3):508–526. 10.1093/sysbio/syaa041 32483631 PMC8048389

[koaf279-B26] Kondrashov FA, Rogozin IB, Wolf YI, Koonin EV. Selection in the evolution of gene duplications. Genome Biol. 2002:3(2):RESEARCH0008. 10.1186/gb-2002-3-2-research0008 11864370 PMC65685

[koaf279-B27] Kuzmin E, Taylor JS, Boone C. Retention of duplicated genes in evolution. Trends Genet. 2022:38(1):59–72. 10.1016/j.tig.2021.06.016 34294428 PMC8678172

[koaf279-B28] Li X, Schmitz RJ. Cis-regulatory dynamics in plant domestication. Trends Genet. 2025:41(11):984–994. 10.1016/j.tig.2025.02.005 40140332

[koaf279-B29] Lu Z, Marand AP, Ricci WA, Ethridge CL, Zhang X, Schmitz RJ. The prevalence, evolution and chromatin signatures of plant regulatory elements. Nat Plants. 2019:5(12):1250–1259. 10.1038/s41477-019-0548-z 31740772

[koaf279-B30] Lynch M, Conery JS. The evolutionary fate and consequences of duplicate genes. Science. 2000:290(5494):1151–1155. 10.1126/science.290.5494.1151 11073452

[koaf279-B31] Marand AP, Eveland AL, Kaufmann K, Springer NM. cis-regulatory elements in plant development, adaptation, and evolution. Annu Rev Plant Biol. 2023:74(1):111–137. 10.1146/annurev-arplant-070122-030236 36608347 PMC9881396

[koaf279-B32] McDonald JMC, Reed RD. Beyond modular enhancers: new questions in cis-regulatory evolution. Trends Ecol Evol. 2024:39(11):1035–1046. 10.1016/j.tree.2024.07.005 39266441

[koaf279-B33] Mendieta JP, Tu X, Jiang D, Yan H, Zhang X, Marand AP, Zhong S, Schmitz RJ. Investigating the cis-regulatory basis of C3 and C4 photosynthesis in grasses at single-cell resolution. Proc Natl Acad Sci U S A. 2024:121(40):e2402781121. 10.1073/pnas.2402781121 39312655 PMC11459142

[koaf279-B34] Ohno S . Evolution by gene duplication. 1970th ed. Berlin, Germany: Springer; 1970. 10.1007/978-3-642-86659-3

[koaf279-B35] Panchy N, Lehti-Shiu M, Shiu S-H. Evolution of gene duplication in plants. Plant Physiol. 2016:171(4):2294–2316. 10.1104/pp.16.00523 27288366 PMC4972278

[koaf279-B36] Qiao X, Li Q, Yin H, Qi K, Li L, Wang R, Zhang S, Paterson AH. Gene duplication and evolution in recurring polyploidization-diploidization cycles in plants. Genome Biol. 2019:20(1):38. 10.1186/s13059-019-1650-2 30791939 PMC6383267

[koaf279-B37] Qiu Y, Van Tay Y, Ruan Y, Adams KL. Divergence of duplicated genes by repeated partitioning of splice forms and subcellular localization. New Phytol. 2020:225(2):1011–1022. 10.1111/nph.16148 31469915

[koaf279-B38] Quinlan AR, Hall IM. BEDTools: a flexible suite of utilities for comparing genomic features. Bioinformatics. 2010:26(6):841–842. 10.1093/bioinformatics/btq033 20110278 PMC2832824

[koaf279-B39] Roulin A, Auer PL, Libault M, Schlueter J, Farmer A, May G, Stacey G, Doerge RW, Jackson SA. The fate of duplicated genes in a polyploid plant genome. Plant J. 2013:73(1):143–153. 10.1111/tpj.12026 22974547

[koaf279-B40] Salman-Minkov A, Sabath N, Mayrose I. Whole-genome duplication as a key factor in crop domestication. Nat Plants. 2016:2:16115. 10.1038/nplants.2016.115 27479829

[koaf279-B41] Schmitz RJ, Grotewold E, Stam M. Cis-regulatory sequences in plants: their importance, discovery, and future challenges. Plant Cell. 2022:34(2):718–741. 10.1093/plcell/koab281 34918159 PMC8824567

[koaf279-B42] Schmutz J, Cannon SB, Schlueter J, Ma J, Mitros T, Nelson W, Hyten DL, Song Q, Thelen JJ, Cheng J, et al Genome sequence of the palaeopolyploid soybean. Nature. 2010:465(7294):120–120. 10.1038/nature08957 20075913

[koaf279-B43] Shi T, Rahmani RS, Gugger PF, Wang M, Li H, Zhang Y, Li Z, Wang Q, Van de Peer Y, Marchal K, et al Distinct expression and methylation patterns for genes with different fates following a single whole-genome duplication in flowering plants. Mol Biol Evol. 2020:37(8):2394–2413. 10.1093/molbev/msaa105 32343808 PMC7403625

[koaf279-B44] Shoemaker RC, Schlueter J, Doyle JJ. Paleopolyploidy and gene duplication in soybean and other legumes. Curr Opin Plant Biol. 2006:9(2):104–109. 10.1016/j.pbi.2006.01.007 16458041

[koaf279-B45] Tiley GP, Ané C, Burleigh JG. Evaluating and characterizing ancient whole-genome duplications in plants with gene count data. Genome Biol Evol. 2016:8(4):1023–1037. 10.1093/gbe/evw058 26988251 PMC4860690

[koaf279-B46] Tran TC, Mähl K, Kappel C, Dakhiya Y, Sampathkumar A, Sicard A, Lenhard M. Altered interactions between cis-regulatory elements partially resolve BLADE-ON-PETIOLE genetic redundancy in *Capsella rubella*. Plant Cell. 2024:36(10):4637–4657. 10.1093/plcell/koae232 39158598 PMC11448885

[koaf279-B47] Wang L, Jia G, Jiang X, Cao S, Chen Z, Song Q. Altered chromatin architecture and gene expression during polyploidization and domestication of soybean. Plant Cell. 2021:33(5):1430–1446. 10.1093/plcell/koab081 33730165 PMC8254482

[koaf279-B48] Wendel JF . Genome evolution in polyploids. Plant Mol Biol. 2000:42(1):225–249. 10.1023/A:1006392424384 10688139

[koaf279-B49] Wittkopp PJ, Kalay G. Cis-regulatory elements: molecular mechanisms and evolutionary processes underlying divergence. Nat Rev Genet. 2012:13(1):59–69. 10.1038/nrg3095 22143240

[koaf279-B50] Wray GA, Hahn MW, Abouheif E, Balhoff JP, Pizer M, Rockman MV, Romano LA. The evolution of transcriptional regulation in eukaryotes. Mol Biol Evol. 2003:20(9):1377–1419. 10.1093/molbev/msg140 12777501

[koaf279-B51] Zhang J . Evolution by gene duplication: an update. Trends Ecol Evol. 2003:18(6):292–298. 10.1016/S0169-5347(03)00033-8

[koaf279-B52] Zhang X, Luo Z, Marand AP, Yan H, Jang H, Bang S, Mendieta JP, Minow MAA, Schmitz RJ. A spatially resolved multi-omic single-cell atlas of soybean development. Cell. 2025:188(2):550–567.e19. 10.1016/j.cell.2024.10.050 39742806 PMC12136577

[koaf279-B53] Zhao M, Zhang B, Lisch D, Ma J. Patterns and consequences of subgenome differentiation provide insights into the nature of paleopolyploidy in plants. Plant Cell. 2017:29(12):2974–2994. 10.1105/tpc.17.00595 29180596 PMC5757279

[koaf279-B54] Zhuang Y, Wang X, Li X, Hu J, Fan L, Landis JB, Cannon SB, Grimwood J, Schmutz J, Jackson SA, et al Phylogenomics of the genus Glycine sheds light on polyploid evolution and life-strategy transition. Nat Plants. 2022:8(3):233–244. 10.1038/s41477-022-01102-4 35288665

