# Supplementary material for: From duplication to divergence: Single-cell insights into transcriptional and cis-regulatory landscapes in soybean
Source: Plant Cell. 2025 Nov 24;37(12):koaf279. doi: 10.1093/plcell/koaf279 (PMC12679596; doi:10.1093/plcell/koaf279)
Supplement: koaf279_Supplementary_Data [file koaf279_supplementary_data.zip › 11192025_supplementary_figures.pdf]

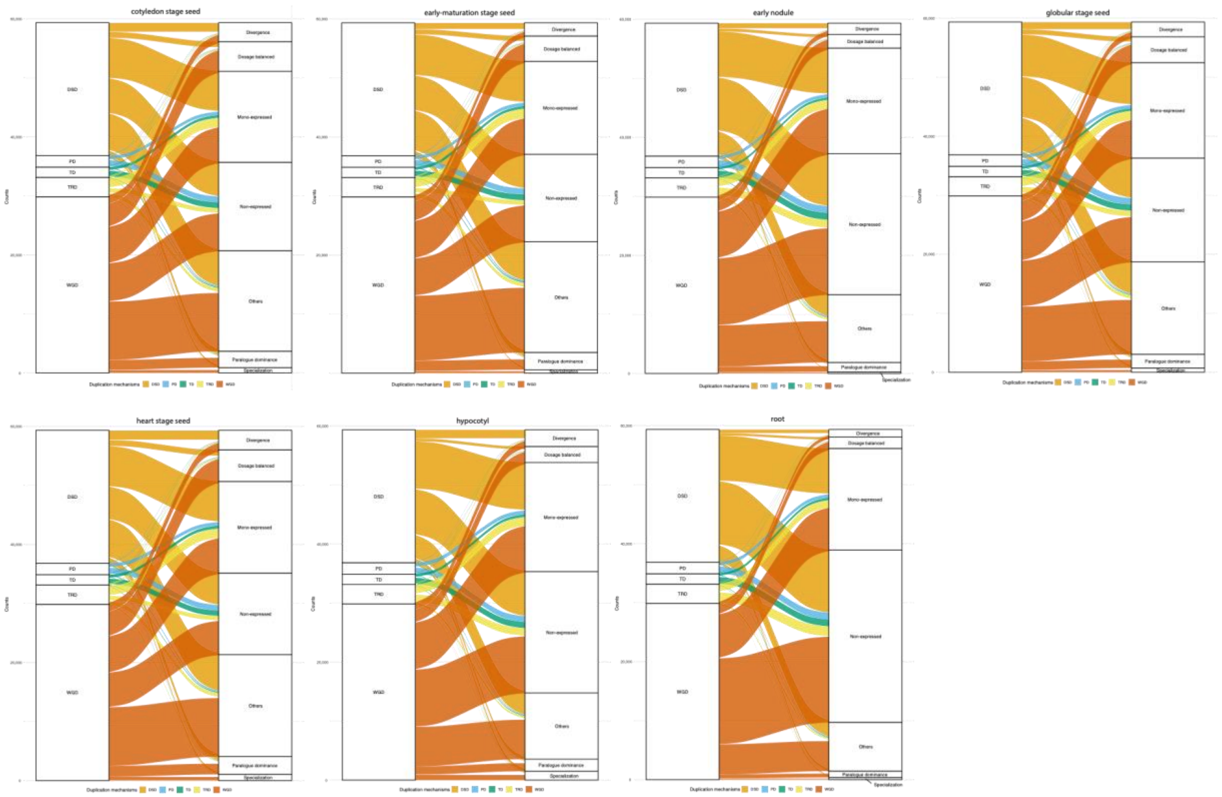

Supplementary Fig. S1. Relationship between duplication mechanisms and within-tissue expression patterns of duplicated gene pairs across different tissues. The left panel categorizes genes based on duplication mechanisms: whole-genome duplication (WGD), tandem duplication (TD), proximal duplication (PD), dispersed duplication (DSD), and transposed duplication (TRD). The right panel represents within-tissue gene expression patterns.

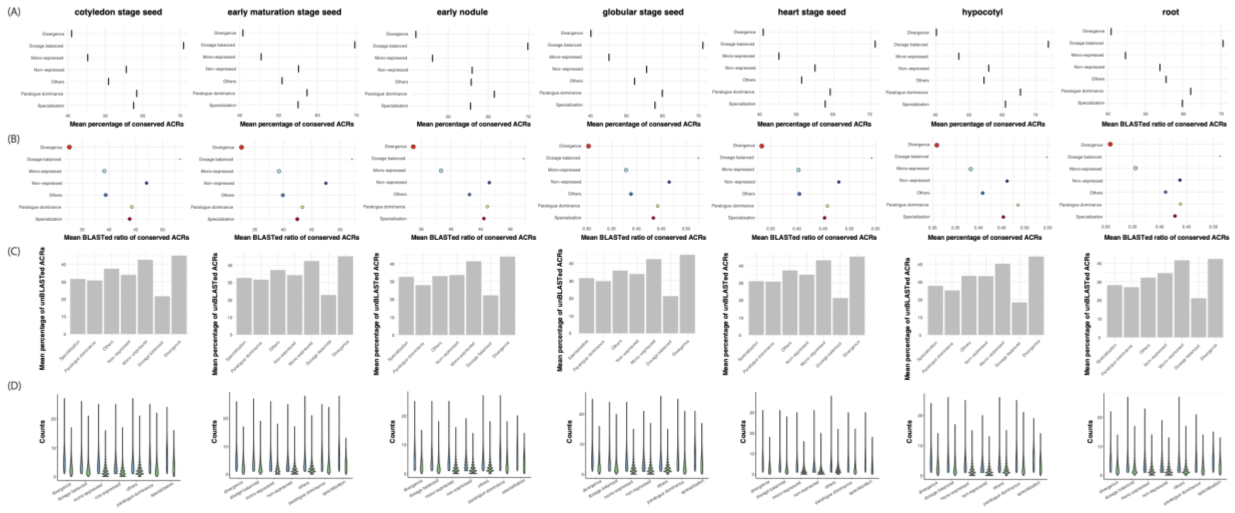

Supplementary Fig. S2. Genetic variation within ACRs associated with gene pairs with different within-tissue expression patterns across different tissues. **(A)** Mean percentage of conserved ACRs (black bars) across gene pairs with different within-tissue expression patterns. **(B)** Mean BLASTed ratio of conserved ACRs across gene pairs with different within-tissue expression patterns. The size of the dots represents the mean mismatch rate of conserved ACRs across gene pairs with different within-tissue expression patterns. **(C)** Mean percentage of ACRs without a corresponding sequence in the reference region (unBLASTed ACRs) across gene pairs with different within-tissue expression patterns. **(D)** Number of ACRs associated with each duplicated gene within the pairs exhibiting different within-tissue expression patterns. Within each gene pair, the gene associated with a higher number of ACRs is classified into the group of large count while the gene associated with fewer ACRs is classified into the group of small count.

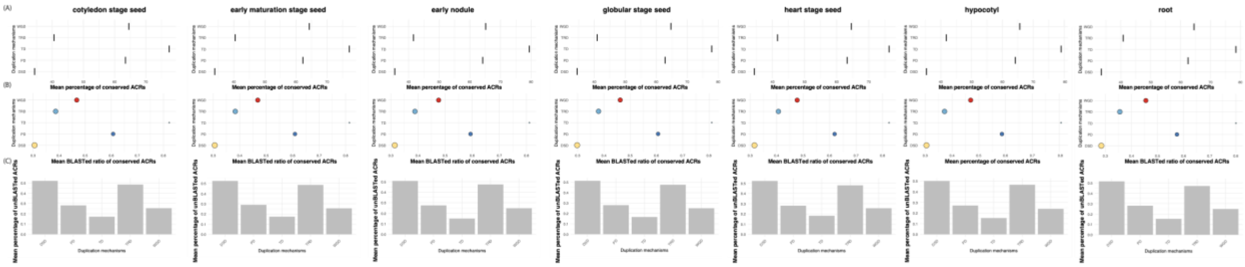

Supplementary Fig. S3. Genetic variation within ACRs associated with gene pairs derived from different duplication mechanisms. **(A)** mean percentage of conserved ACRs (black bars) across gene pairs derived from different duplication mechanisms. **(B)** mean BLASTed ratio of conserved ACRs across gene pairs derived from different duplication mechanisms. The size of the dots represents the mean mismatch rate of conserved ACRs across gene pairs derived from different duplication mechanisms. **(C)** mean percentage of ACRs without a corresponding sequence in the reference region (unBLASTed ACRs) across gene pairs derived from different duplication mechanisms. WGD: whole-genome duplication; TD: tandem duplication; PD: proximal duplication; DSD: dispersed duplication; and TRD: transposed duplication.

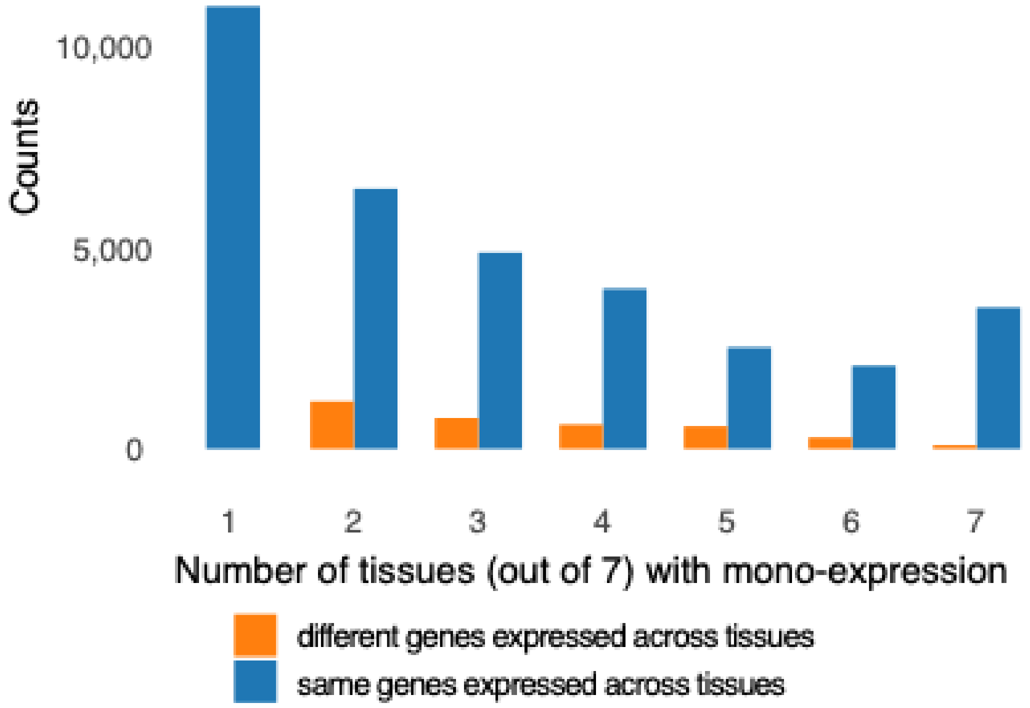

Supplementary Fig. S4. Histogram showing counts of mono-expressed gene pairs, comparing whether the same or different genes are expressed across tissues. Mono-

expressed gene pairs typically exhibit a consistent pattern in which the same gene is expressed while the other remains non-expressed across multiple tissues.

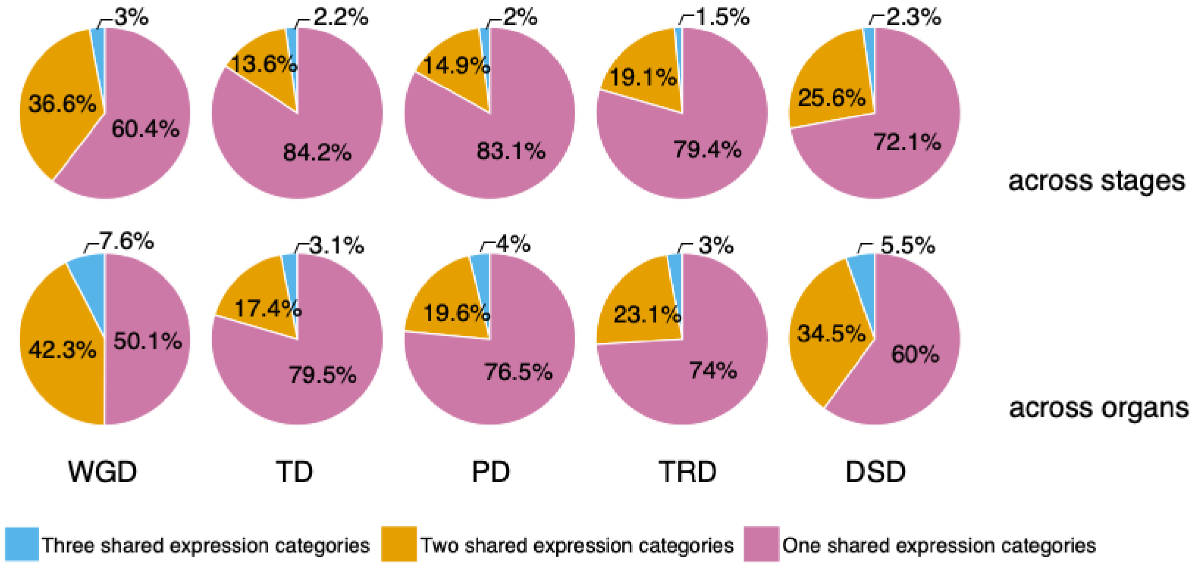

Supplementary Fig. S5. Pie charts of gene pairs from different duplication mechanisms, with each chart showing the percentages of duplicated gene pairs grouped by the number of shared expression categories across developmental stages or organs. WGD: whole-genome duplication; TD: tandem duplication; PD: proximal duplication; DSD: dispersed duplication; and TRD: transposed duplication.

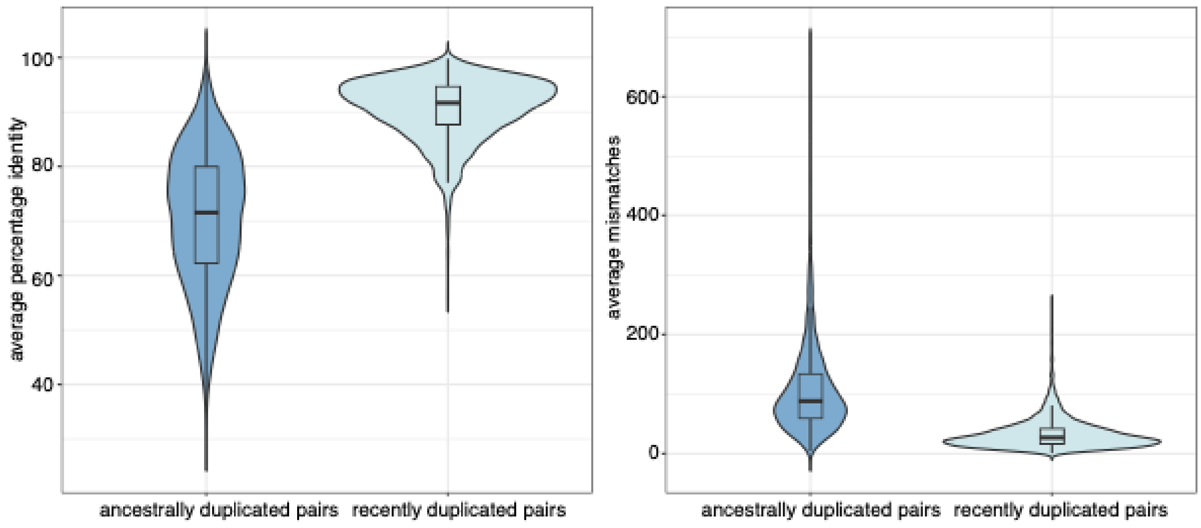

Supplementary Fig. S6. Comparison of protein sequence similarity between ancestrally and recently duplicated gene pairs.

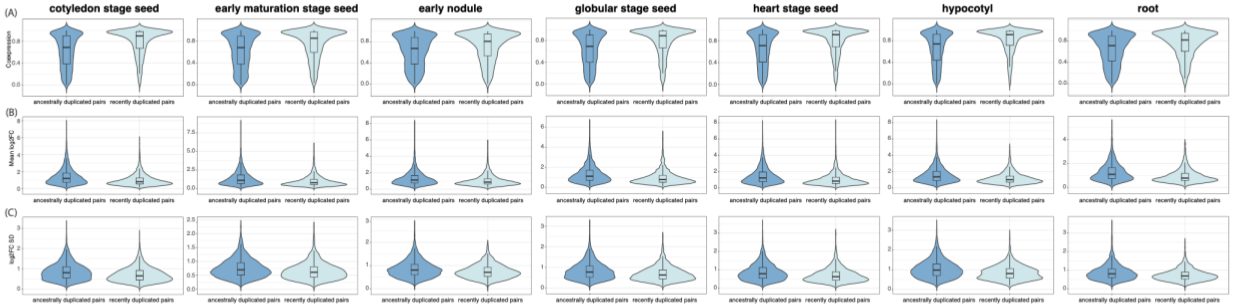

Supplementary Fig. S7. Comparison of expression divergence between ancestrally and recently duplicated gene pairs within each individual tissue. (A) coexpression values. (B) mean log2FC and (C) SD of log2FC. FC: Fold change; and SD: standard deviation.

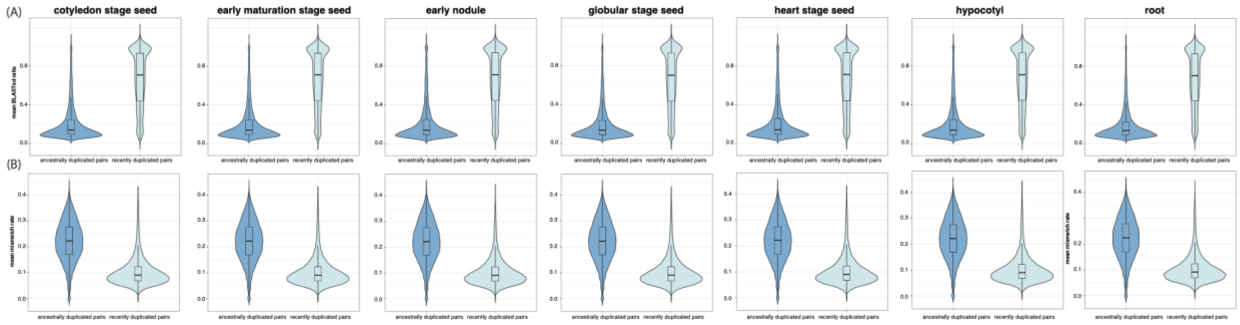

Supplementary Fig. S8. Comparison of sequence divergence of ACRs associated with ancestrally and recently duplicated gene pairs within each individual tissue. (A) mean BLASTed ratio. (B) mean mismatch rate.

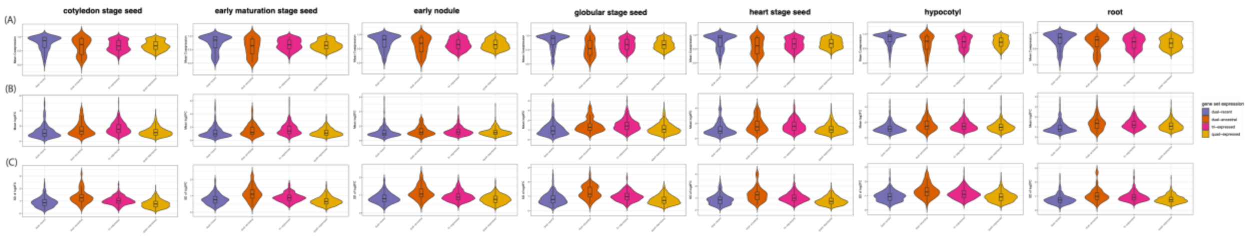

Supplementary Fig. S9. Transcriptional divergence of four-gene sets within each individual tissue measured both by the number of expressed genes per set and their expression correlations. (A) coexpression value. (B) mean log2FC and (C) SD of log2FC. FC: Fold change; and SD: standard deviation.

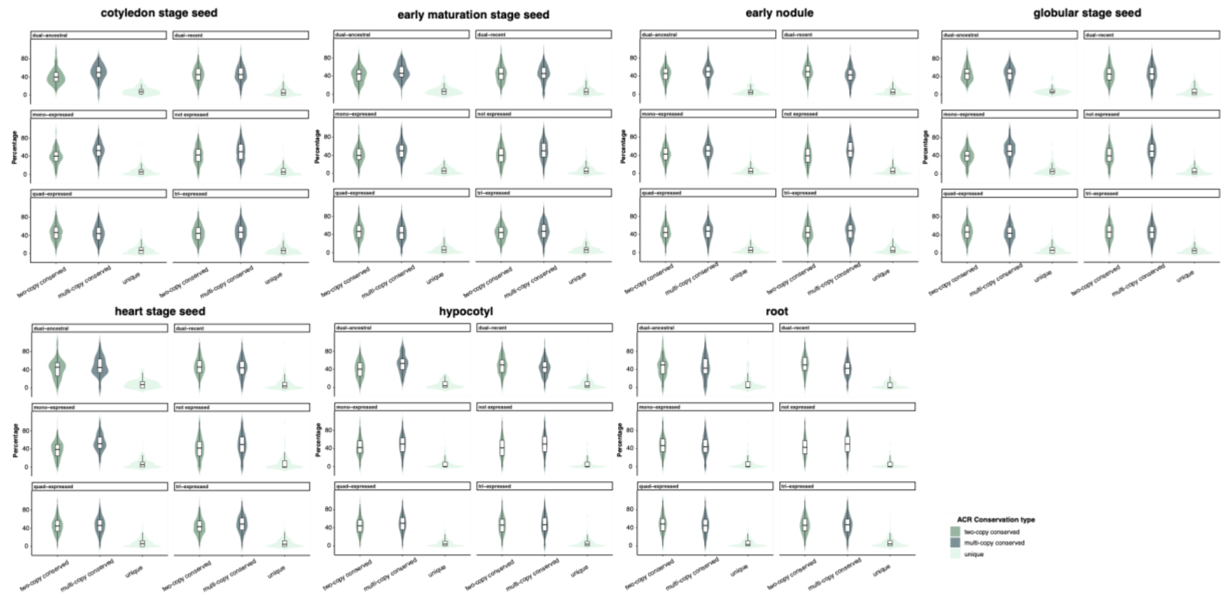

Supplementary Fig. S10. Percentages of each ACR conservation type identified in four-gene sets exhibiting different expression patterns within each individual tissue.

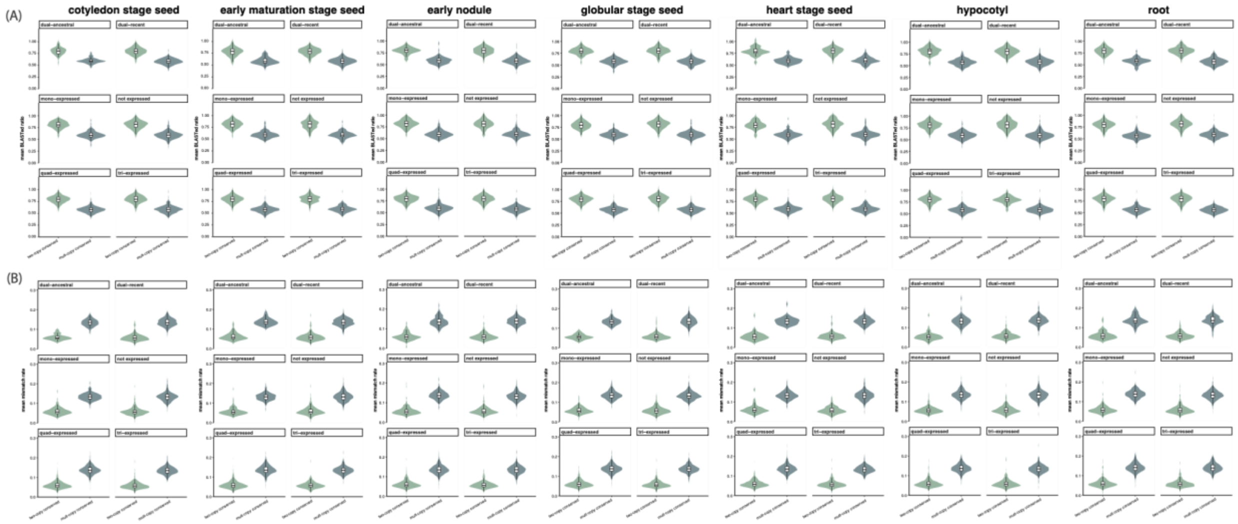

Supplementary Fig. S11. Sequence similarity of two-copy conserved versus multi-copy conserved ACRs associated with four-gene sets exhibiting distinct expression patterns within each individual tissue. **(A)** mean BLASTed ratio, and **(B)** mean mismatch rate.

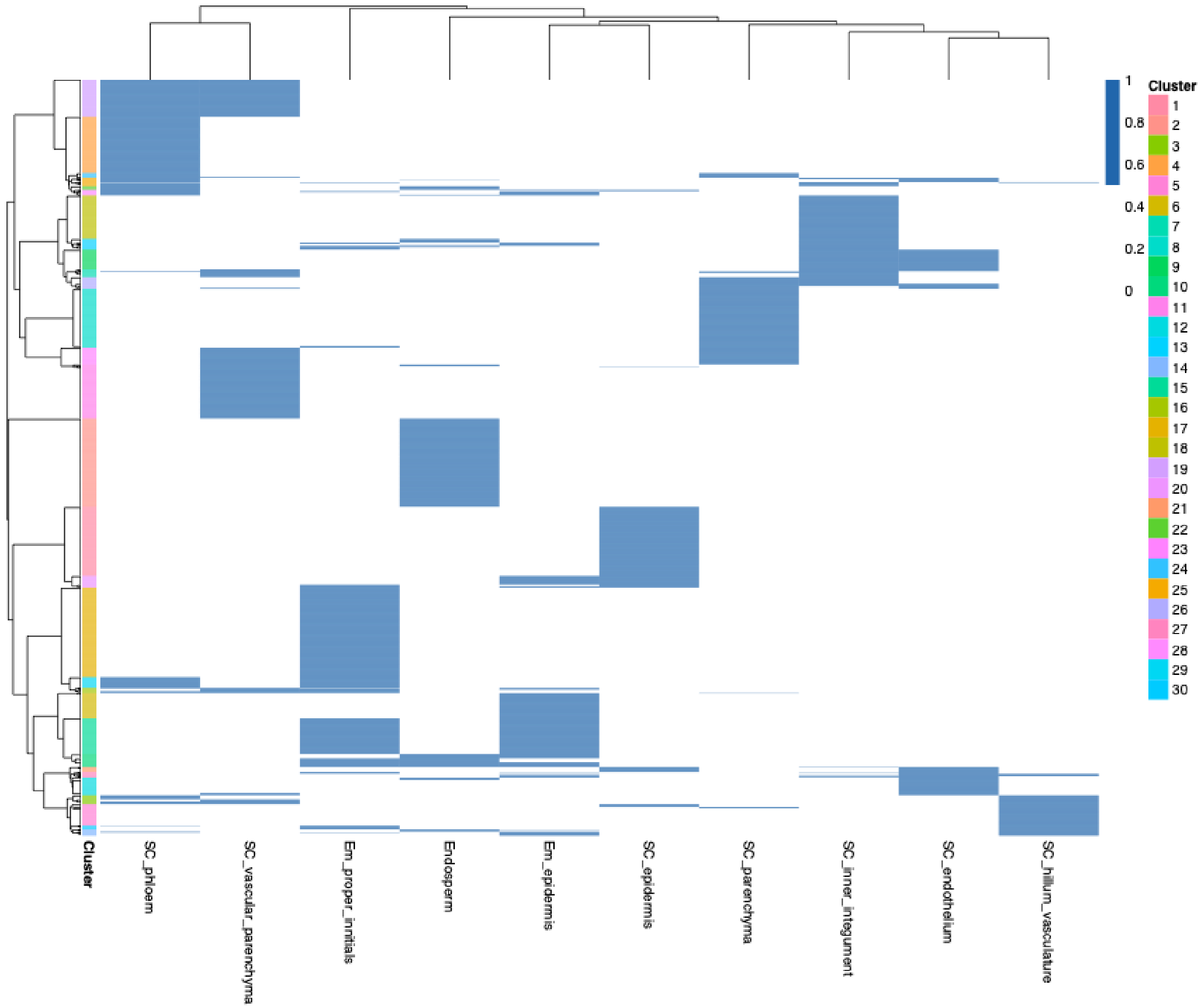

Supplementary Fig. S12. Heatmap of the clusters identified across four gene sets, each containing at least one gene with cell-type-specific expression in cotyledon stage seed. SC: Seed Coat. Em: Embryo.

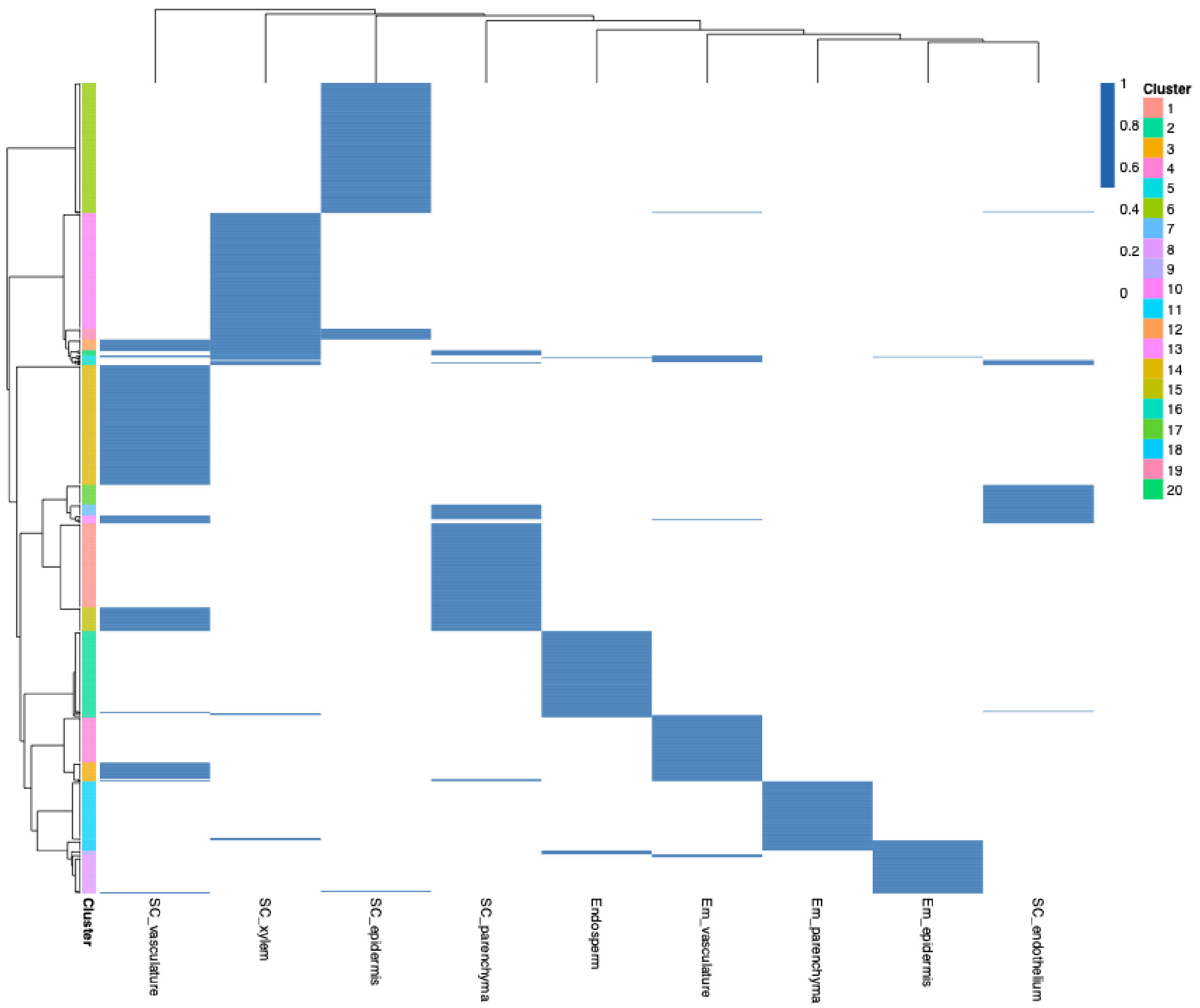

Supplementary Fig. S13. Heatmap of the clusters identified across four gene sets, each containing at least one gene with cell-type-specific expression in early maturation stage seed. SC: Seed Coat. Em: Embryo.

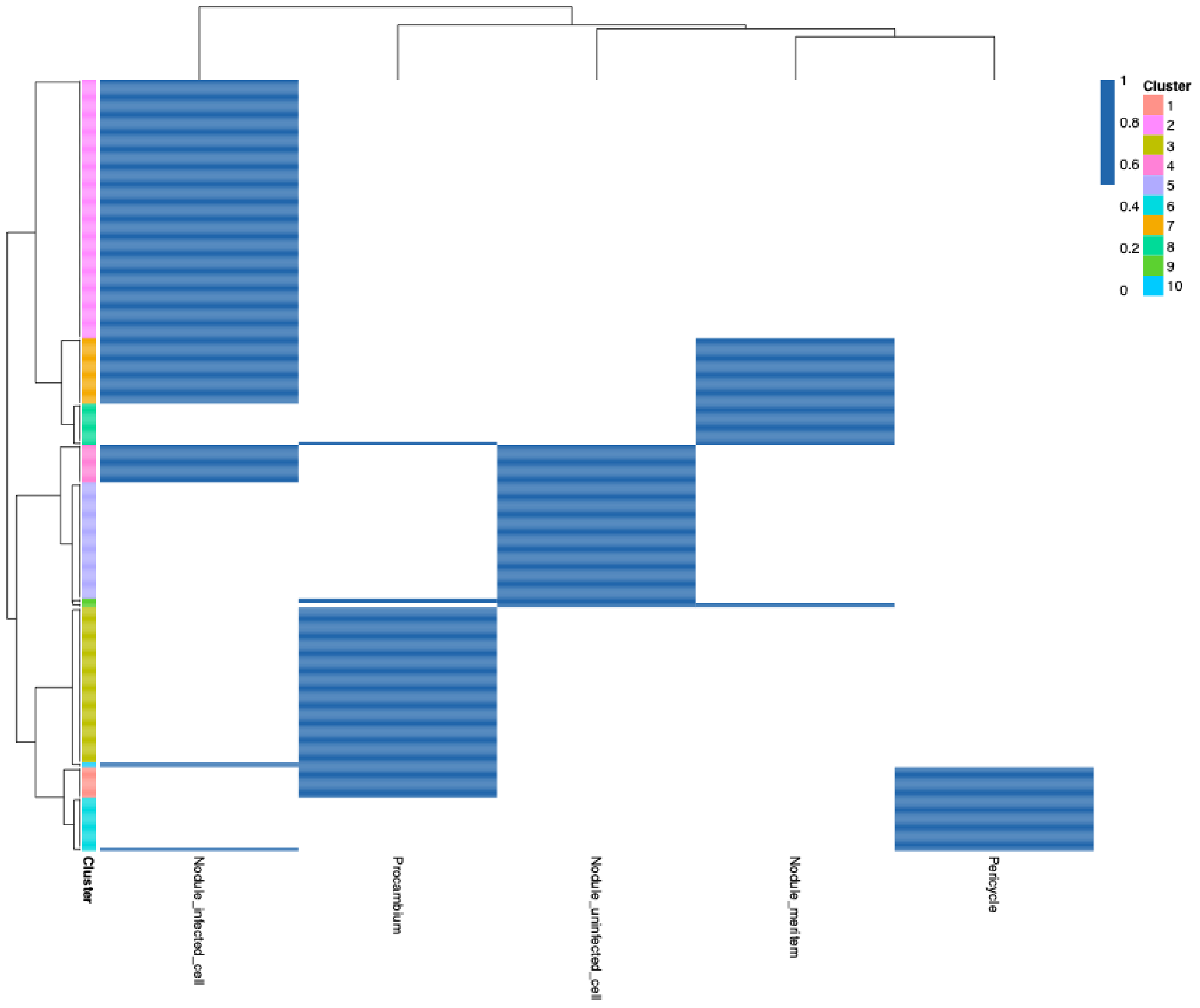

Supplementary Fig. S14. Heatmap of the clusters identified across four gene sets, each containing at least one gene with cell-type-specific expression in nodule.

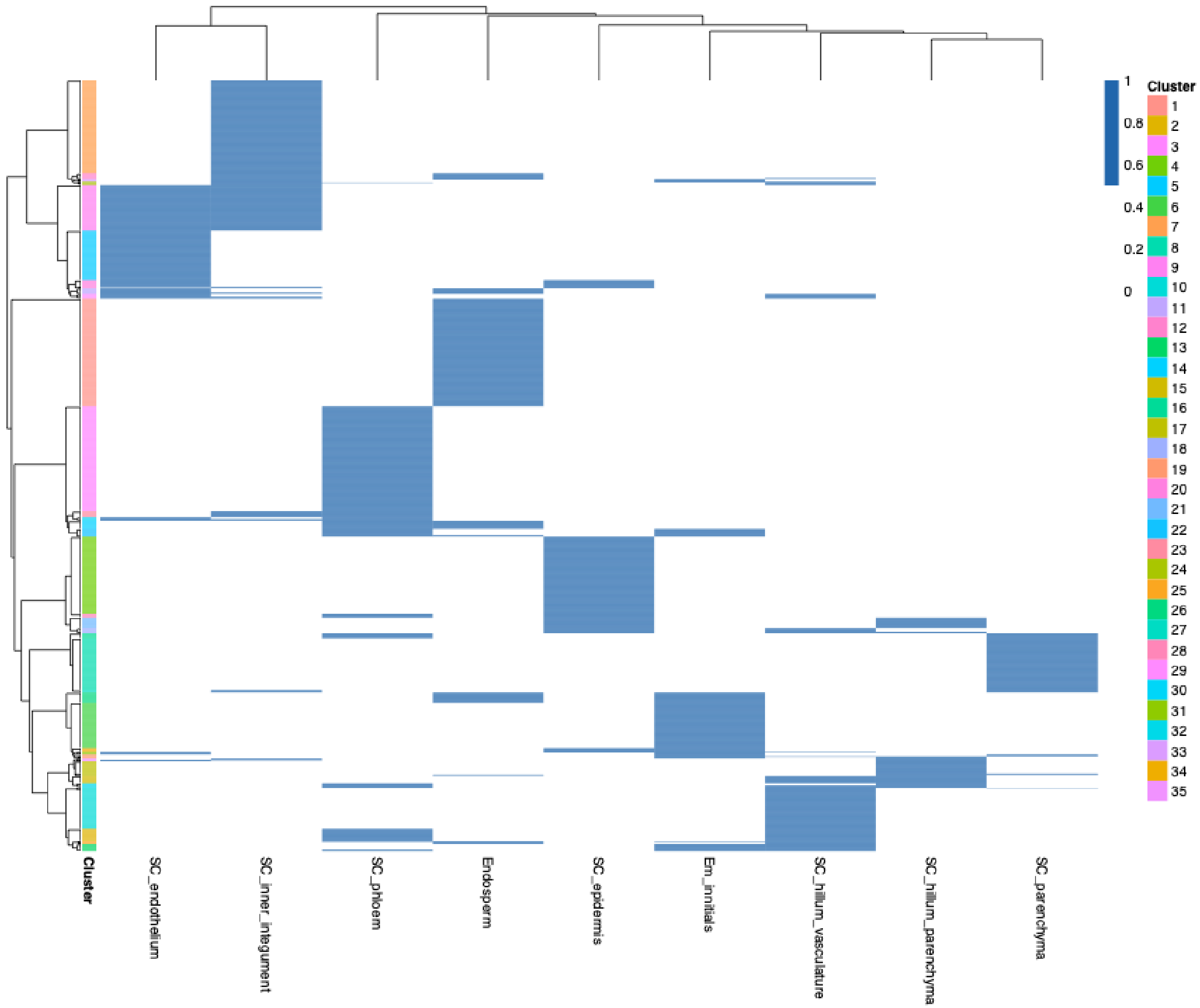

Supplementary Fig. S15. Heatmap of the clusters identified across four gene sets, each containing at least one gene with cell-type-specific expression in globular stage seed. SC: Seed Coat. Em: Embryo.

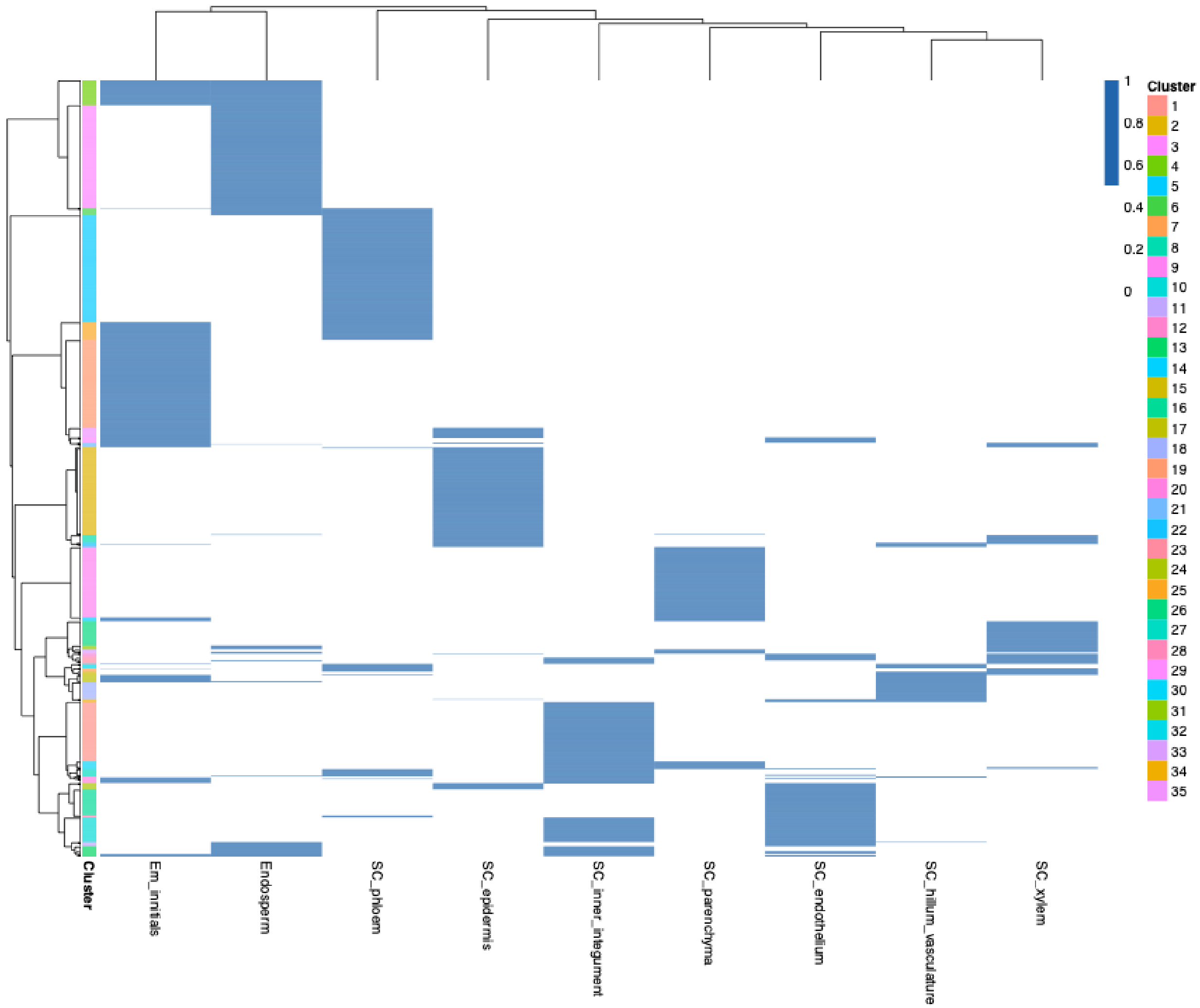

Supplementary Fig. S16. Heatmap of the clusters identified across four gene sets, each containing at least one gene with cell-type-specific expression in heart stage seed. SC: Seed Coat. Em: Embryo.

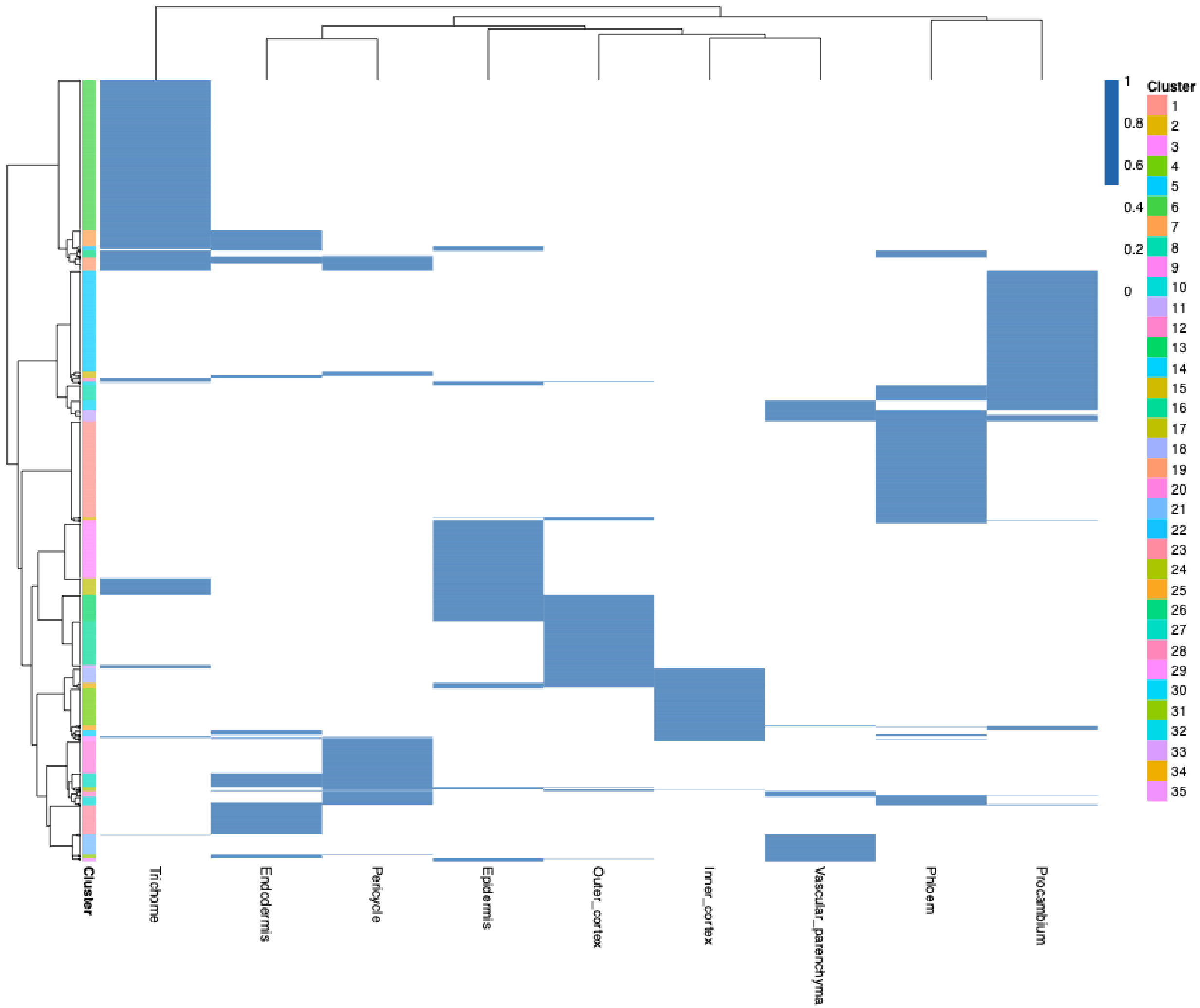

Supplementary Fig. S17. Heatmap of the clusters identified across four gene sets, each containing at least one gene with cell-type-specific expression in hypocotyl.

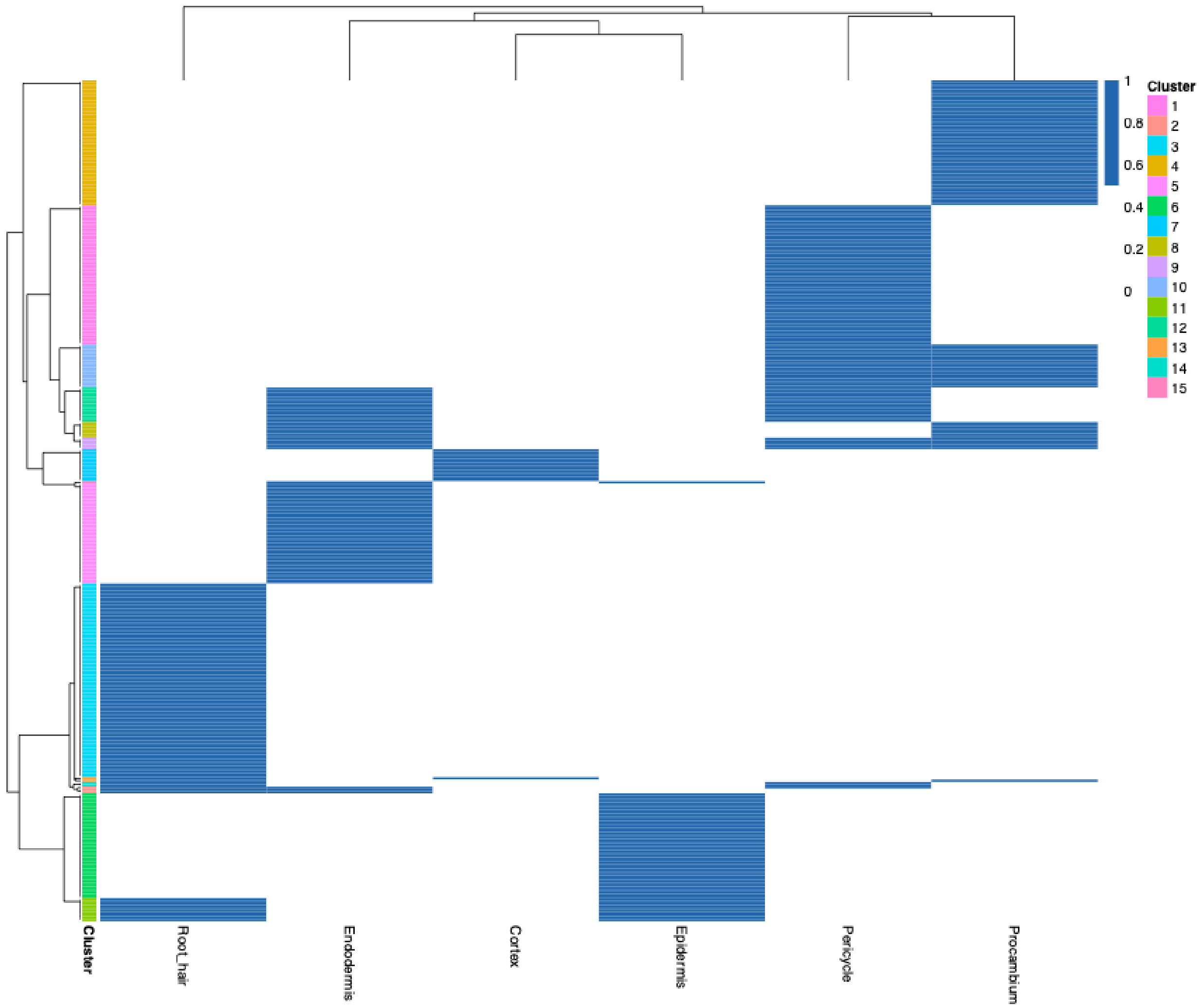

Supplementary Fig. S18. Heatmap of the clusters identified across four gene sets, each containing at least one gene with cell-type-specific expression in root.

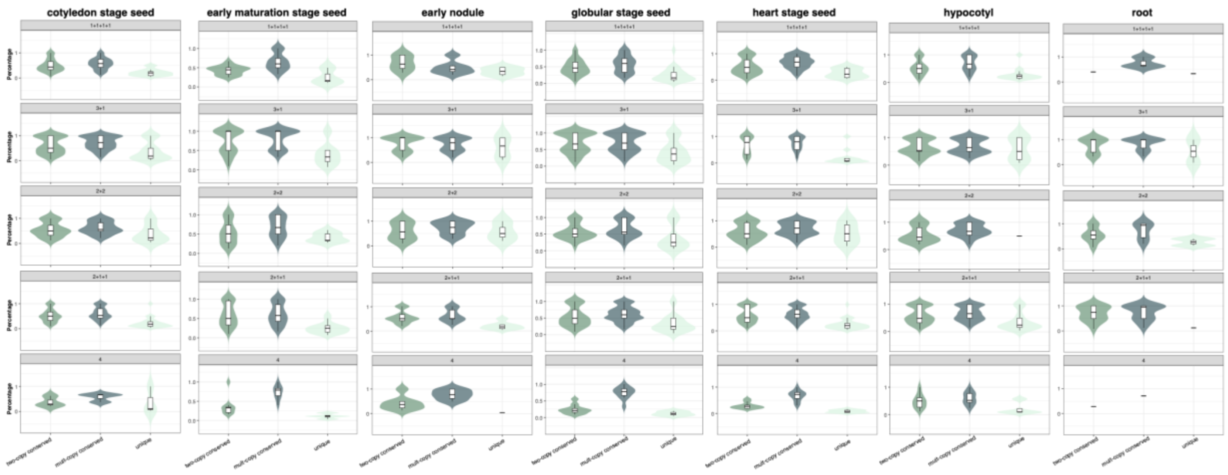

Supplementary Fig. S19. Percentages of each ACR conservation type identified in cell-type-specific ACRs associated with four-gene sets exhibiting different evolutionary trajectories of cell-type-specific expression.

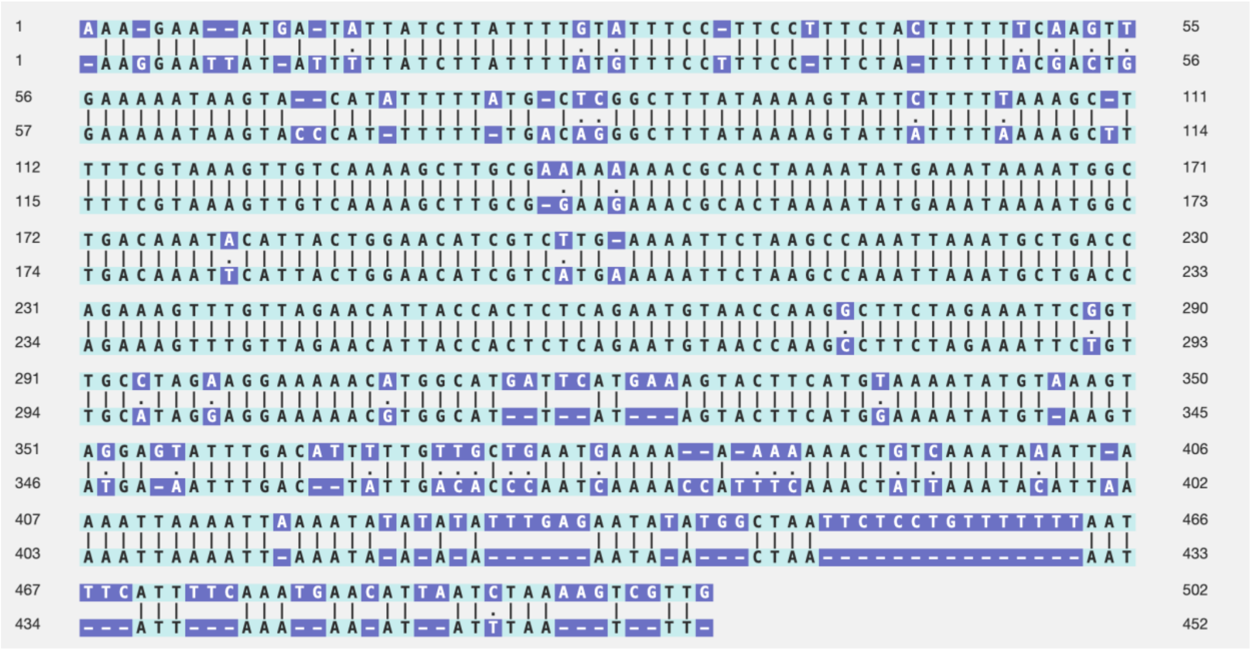

Supplementary Fig. S20. Sequence alignment of the two ctACRs associated with recently duplicated gene pairs that exhibit seed coat epidermis–specific expression patterns (as shown in Fig. 6C).
